# Parallel functional architectures within a single dendritic tree

**DOI:** 10.1016/j.celrep.2023.112386

**Published:** 2023-04-13

**Authors:** Young Joon Kim, Balázs B. Ujfalussy, Mátê Lengyel

**Affiliations:** 1 Computational and Biological Learning Lab, Department of Engineering, University of Cambridge, Cambridge, UK; 2 Harvard Medical School, Boston, MA, USA; 3 Laboratory of Biological Computation, Institute of Experimental Medicine, Budapest, Hungary

## Abstract

The input-output transformation of individual neurons is a key building block of neural circuit dynamics. While previous models of this transformation vary widely in their complexity, they all describe the underlying functional architecture as unitary, such that each synaptic input makes a single contribution to the neuronal response. Here, we show that the input-output transformation of CA1 pyramidal cells is instead best captured by two distinct functional architectures operating in parallel. We used statistically principled methods to fit flexible, yet interpretable, models of the transformation of input spikes into the somatic “output” voltage and to automatically select among alternative functional architectures. With dendritic Na^+^ channels blocked, responses are accurately captured by a single static and global nonlinearity. In contrast, dendritic Na^+^-dependent integration requires a functional architecture with multiple dynamic nonlinearities and clustered connectivity. These two architectures incorporate distinct morphological and biophysical properties of the neuron and its synaptic organization.

## Graphical abstract

**Graphic abstract:**
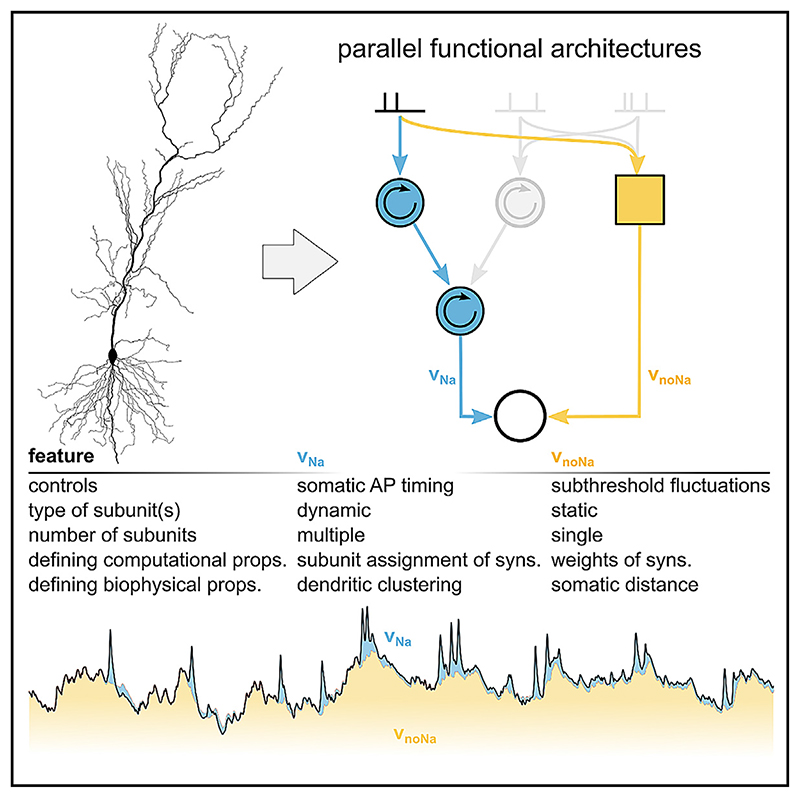

## Introduction

Under *in vivo* conditions, cortical neurons receive thousands of synaptic inputs and integrate them through diverse computations distributed across the dendritic arbor.^1,2^ These integrative processes determine how spatiotemporal patterns of “input” spikes impinging the cell are transformed into its time-varying somatic membrane potential “output.” Accurately characterizing this input-output transformation in individual neurons is a key step toward understanding the collective dynamics of neural circuits.^3,4^

**Figure 1:**
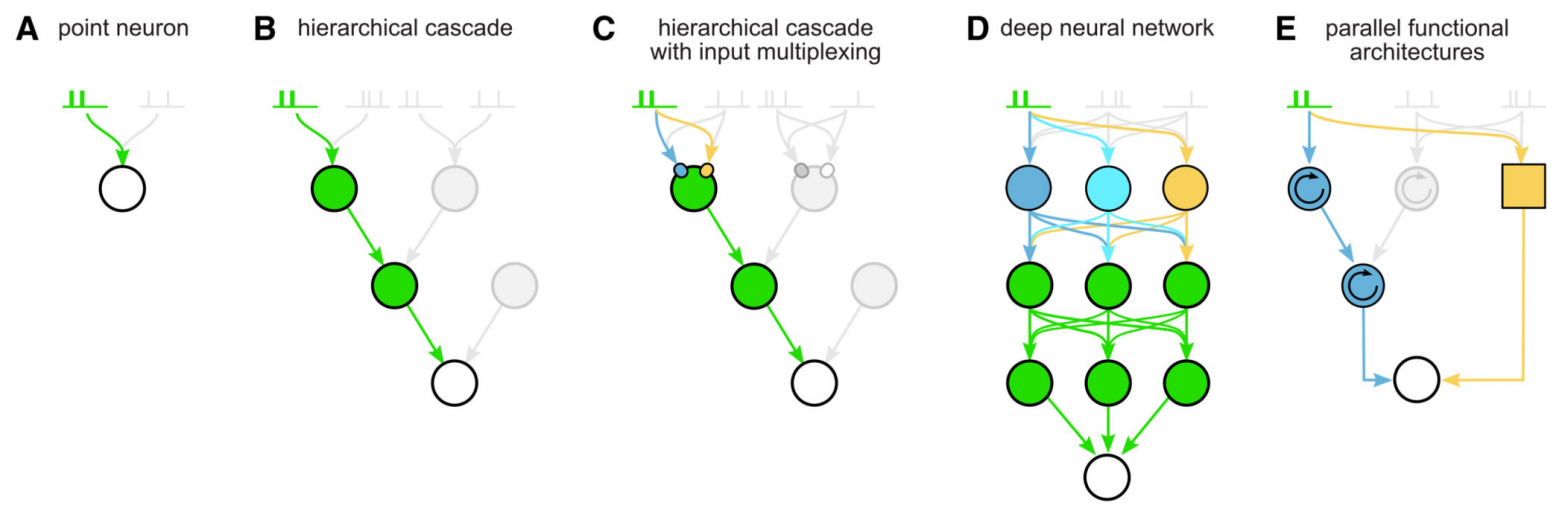
Comparison of computational models of the neuronal input-output transformation Each shape (circle or square) represents a functional “subunit,” performing an elementary operation (e.g., summation followed by a sigmoid nonlinearity) on the inputs (incoming arrows, from spikes arriving via afferent synapses or from other subunits) to produce its output (outgoing arrow). The output of the cell is produced by the bottom subunit (open circle). Identical shapes indicate subunits performing similar operations. An example input spike train is highlighted in green, and the pathway processing this input is highlighted in colors. Splitting and mixing of colors indicates divergence and convergence of this pathway: parts conveying distinct contributions of this input are shown in separate colors (blue and yellow), and parts conveying mixed contributions are shown in green. (A) Point neuron model with no hierarchical processing.^6–8^ (B) Hierarchical cascade: each spike train is processed by only a single pathway.^9–2^ (C) Hierarchical cascade with input multiplexing.^9^ Each spike train is processed by multiple rapidly converging pathways that are kept separate only at the level of inputs and use the same type of elementary operation. (D) Deep neural network (DNN).^13,14^ Each spike train is processed by multiple rapidly converging pathways that typically diverge and converge in each step, always using the same type of elementary operation. (E) Parallel architectures (this study). The processing pathways of the same spike train converge only in the last step of the hierarchy, and these pathways belong to different functional architectures using different elementary operations (circles versus square).

Abstract mathematical models developed to capture the input-output transformations of cortical neurons vary widely in their complexity.^5^ These models can be operationalized as a set of “subunits” (Figure 1, circles and square) connected by directed edges (Figure 1, arrows between shapes). Each subunit receives some subset of the cell’s synaptic inputs (Figure 1, spike trains on top) as well as input from other subunits (as dictated by its incoming connections) and performs some elementary mathematical operation on these inputs. The result of this mathematical operation is relayed to other subunits (as dictated by the subunit’s outgoing connections) or forms the predicted output of the cell (Figure 1, bottom-most subunit). Existing models differ in their functional architecture, as defined by the number and connectivity of subunits and the form of mathematical operations used in those subunits (Figures 1A–1D). Some models perform (either based on simplifying assumptions^6–8^ or supported by data-driven analysis^9^) a fundamentally linear integration of all synaptic inputs arriving at the cell, followed by only a single, global nonlinearity. In other words, they use a single, so-called linear-nonlinear subunit (Figure 1A). Other models posit that synaptic inputs are divided into a limited number of functional clusters, such that inputs within each cluster are processed by a local linear-nonlinear processing subunit, and a hierarchical cascade of such subunits determines somatic output^9–12^ (Figures 1B and 1C). Yet more complex models characterize the input-output transformations of a single neuron as equivalent to a multilayer deep neural network (DNN) with nearly a thousand linear-nonlinear (sub)units^13,14^ (Figure 1D).

Despite the apparent diversity of proposals for the mathematical form of neuronal input-output transformations, previous models all share a fundamental feature: they characterize this transformation by a functional architecture that can be considered “unitary.” A unitary architecture is defined by specific constraints on the connectivity and type of its subunits. The constraints on connectivity can be best understood by following the sequence of subunits that any particular synaptic input passes through before reaching the output subunit (Figures 1A–1D, colored pathways originating from green spike train). This sequence consists of either a single pathway (as in sparsely connected cascade models^10–12^; Figures 1A and 1B, green pathway) or multiple processing pathways (e.g., multiplexed cascades^9^or densely connected DNNs^13,14^; Figures 1C and 1D, green spike train feeding blue and yellow pathways). However, even when multiple pathways exist in a unitary architecture, they converge rapidly, such that their contributions to the final output of the model cannot be dissected (Figures 1C and 1D, blue and yellow pathways mixing into green after one step). The other constraint, in both single- and multiple-pathway models, is that all subunits perform fundamentally the same type of mathematical operation (typically a simple linear-nonlinear transformation; Figures 1A–1D, circles). Therefore, these models may suggest that all the biophysical complexity underlying the input-output mapping of neurons can be summarized by each synaptic input making only one kind of contribution to the output of the cell.

However, dendritic arbors typically harbor a diverse set of biophysical integrative mechanisms with a broad range of distinct time scales and input sensitivities. At one end of the spectrum, fast dendritic Na^+^ spikes are triggered by highly synchronized inputs arriving within a few milliseconds onto a short dendritic segment.^15^ Although dendritic Na^+^ spikes can contribute to the precise timing of somatic action potentials,^16–21^ the sharp nonlinearity of the sodium channel activation makes them sensitive to small fluctuations in the inputs and their propagation unreliable.^19,22,23^ At the other end of the spectrum are NMDA receptor-mediated plateau potentials^24^ that are more robust to spatiotemporal variations in the inputs.^25–27^ Although the propagation of these events to the soma is not actively maintained, they can still substantially contribute to somatic depolarization and to robust neuronal tuning under *in vivo* conditions.^21,28^

The diversity of dendritic mechanisms suggests that a single, unitary functional architecture with a single type of subunit may be insufficient to accurately describe the neuronal input-output transformation. Instead, it motivates an approach loosely analogous to the well-known Fourier decomposition of a potentially complex transformation into a sum of elementary transformations operating at distinct frequencies in parallel. Specifically, it suggests that the effects of dendritic mechanisms operating at different time scales may be best understood by distinct functional architectures, with different types of subunits (Figure 1E, circles versus square), operating in parallel and converging only in the last summation step (Figure 1E, blue and yellow pathways mixing only at the bottom). This way, even a single synaptic input could make several distinct contributions to the cell’s output, one contribution via each architecture.

While the existing empirical evidence is broadly suggestive of parallel architectures with different types of subunits,^29^ there remain several key issues unresolved. First, most of this evidence was obtained *in vitro,* while understanding dendritic integration under *in vivo* conditions presents unique challenges^9^ because natural synaptic stimuli may contain multiple input patterns at any given time, and different dendritic mechanisms could interact with one another in complex ways.^26,30^ This may potentially render the effective contributions of biophysically distinct mechanisms to the cell’s output difficult to dissect. As a result, such intermingled contributions may be best described by functional subunits that are largely similar, or indeed are not even separable, which would thus still be consistent with a unitary functional architecture. Second, without a formal mathematical analysis, earlier studies did not distinguish whether the effects of these distinct mechanisms converge early in the functional architecture of the cell, again consistent with a unitary architecture, or they converge late, thus giving rise to genuinely parallel architectures.

To study the functional architecture of neuronal input-output transformations under *in vivo*-like conditions, and in particular to establish formally whether its underlying architecture is unitary or parallel, we used a model-based approach that allowed us to rigorously characterize this transformation in a data-driven way. For this, we first extended previously developed methods in which subunits had only static nonlinearities^9–11^ by incorporating subunits capturing arbitrary nonlinear dynamics. Second, we fit all the parameters of the model, including those parameterizing the dynamical nonlinearities, as well as the architecture of the model in a data-driven way, with minimal prior assumptions. Using these methods, we characterized the input-output transformation of a hippocampal CA1 pyramidal cell during the “theta state.”^31^ We found that predicting the contribution of dendritic Na^+^ channels to the output of the cell (v_Na_) required a functional architecture fundamentally distinct from predicting the contributions of all other dendritic signals (v_noNa_). Specifically, while v_noNa_ could be accurately predicted using a single subunit with a static nonlinearity, precisely capturing vNa required a larger number of dendritic subunits equipped with nonlinear dynamics and connected into an architecture that appropriately reflected the clustering of synaptic inputs onto the dendritic tree. These results suggest the presence of at least two distinct functional architectures within the same dendritic tree, operating in parallel.

## Results

We chose hippocampal CA1 pyramidal cells as our test bed to study the neuronal input-output transformation, as this is a paradigmatic principal cell type in the cortex with validated biophysical models^32,33^ and extensively characterized input statistics *in vivo* in behaving animals.^34,35^ Briefly, we used a CA1 biophysical model neuron (STAR Methods) that was tuned to reproduce various somatic and dendritic integrative properties under *in vitro* conditions^33^ (Figure S1). These included the generation and propagation of Na^+^ spikes along the dendritic arbor^26^ (Figure S1B), the amplitude distribution of synaptic responses,^36^ the nonlinear integration of inputs via NMDA receptors^26^ (NMDARs; Figures S1A and S1C), and A-type K^+^ channels for limiting dendritic excitability.^37^ To test the robustness of our analyses, we used a range of NMDAR and dendritic Na^+^ conductances that produced realistic patterns of input integration (Figures S1D and S1E).

**Figure 2:**
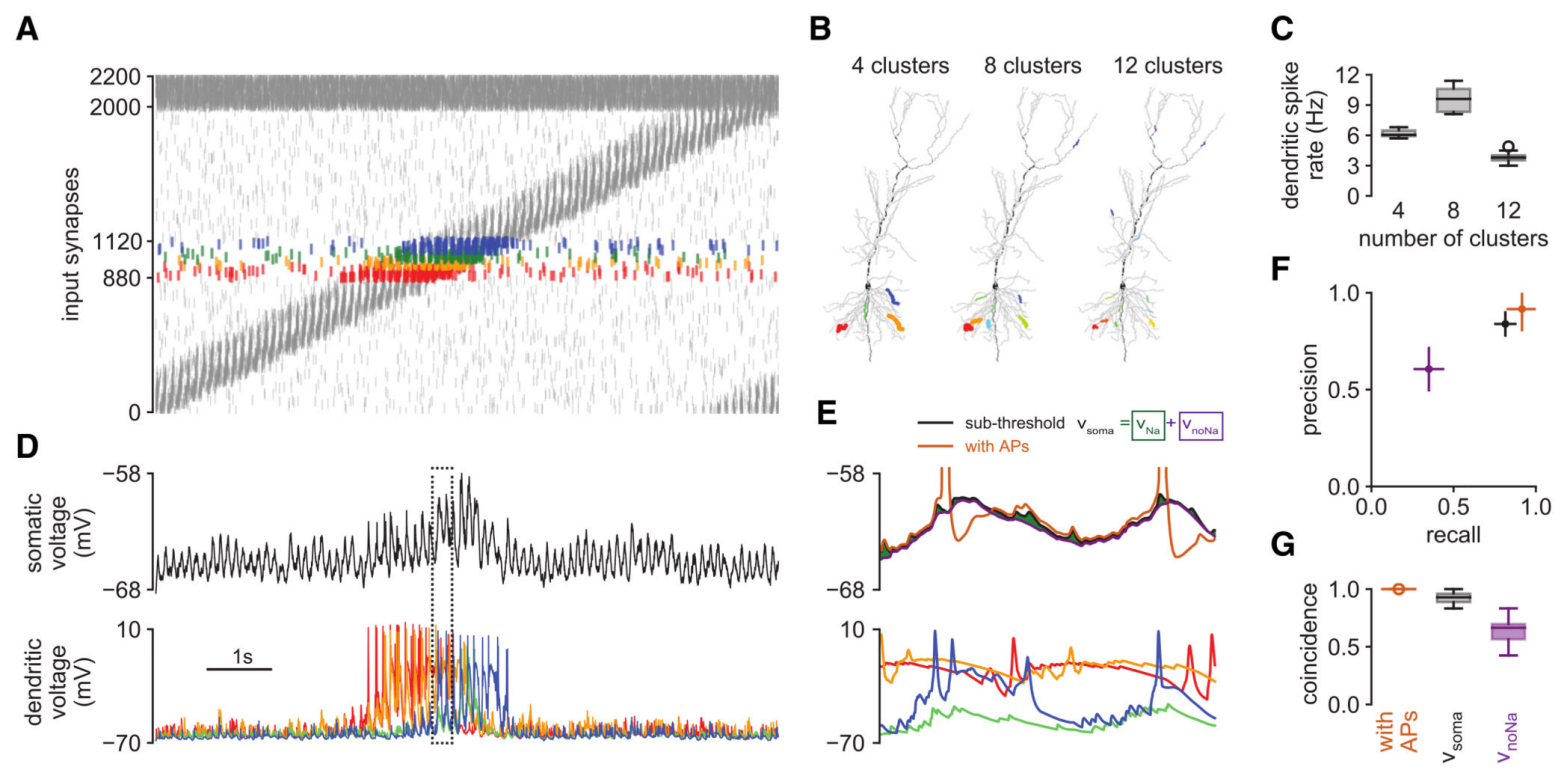
Dendritic Na^+^ spikes are critical for action potential timing in a biophysical CA1 model (A) Spike raster plots for one 10-s trial showing spikes at 2,000 excitatory (bottom) and 200 inhibitory (top) input synapses. Two hundred forty excitatory synapses (colors) are grouped into synaptic clusters of various sizes (four clusters are shown here, see also B). (B) Schematics showing the locations of input synapses along the dendritic tree for “background” excitatory (gray) and clustered excitatory (colors) inputs for three different arrangements of the 240 clustered synapses. Dot sizes are proportional to the frequency of Na^+^ spikes in the target branch. (C) Frequency of dendritic Na^+^ spikes for the three cluster arrangements (shown in B). (D) Dendritic (bottom, colors as in A and B, left) and subthreshold somatic (top, black) membrane potential traces in the four-cluster arrangement during the trial shown in (A). Dendritic traces were recorded at the sites of clustered excitatory inputs. Dotted rectangle shows period magnified in (E). (E) Close-up view of the dendritic (bottom, colors) and subthreshold somatic membrane potential traces (top, black, v_soma_) from (D). The subthreshold somatic membrane potential after blocking dendritic Na^+^ channels is shown in purple (v_noNa_), and its difference from v_soma_, i.e., the effective contribution of dendritic Na^+^channels to the subthreshold somatic membrane potential, is shown in green (v_Na_). For reference, the somatic membrane potential including somatic action potentials is also shown (orange). (F) Precision versus recall of somatic action potential prediction using the subthreshold somatic membrane potential with (black) or without (purple) dendritic Na^+^spikes. For reference, performance using the full somatic membrane potential is also shown (orange). (G) Same as (F) but quantifying decoding accuracy with the spike coincidence metric of Naud et al.^43^ Boxplots in (C) and (G) show median (horizontal line), 25th and 75th percentiles (box), ±1.5 interquartile ranges (whiskers), and outliers (circles) across 20 trials. Crosshairs in (F) show mean ±1 SD along each axis across 20 trials. Data in (F) and (G) are from the four-cluster arrangement. See also Figures S1 and S2.

We stimulated the biophysical model with synaptic input patterns modeled to represent *in vivo*-like conditions.^33^ Specifically, we used 2,000 excitatory and 200 inhibitory inputs with spiking patterns expected to occur in an animal navigating a one-dimensional environment (a circular track). Excitatory inputs showed place cell-like activity, with location-dependent firing rate tuning curves and spike timings exhibiting theta-phase precession,^38,39^ while inhibitory inputs were not specifically tuned to location and were modulated only by the phase of the ongoing theta oscillation^40,41^ (Figure 2A). Of the 2,000 excitatory inputs, 240 of them, with place fields covering a continuous segment of the track (Figure 2A, colors), were grouped into synaptic clusters^42^ (Figure 2B), of which we varied the number between 4 and 12. Synapses within a cluster were co-located on the same dendritic branch, but the clusters themselves, as well as the remaining “background” synapses, were distributed randomly throughout the dendritic tree (Figure 2B). Critically, the clustering of inputs allowed the generation of dendritic spikes (Figure 2C).

### Dendritic spikes control somatic action potential output

The somatic subthreshold membrane potential of this model showed realistic fluctuations, characterized by strong theta modulation,^32,44^ and elevated levels within a localized region of the environment^32^(Figure 2D, top). Place tuning was accompanied by dendritic spikes generated in branches receiving clustered inputs^45^ (Figure 2D, bottom). These dendritic spikes appeared as spikelets in the somatic voltage^26^ (Figure 2E, compare black trace at top with colored traces at bottom). Although spikelets were small in magnitude, they seemed to have a disproportionately strong control over the timing of somatic action potentials (after “unblocking” somatic voltagedependent Na^+^ channels in our simulations; Figure 2E, top orange). To quantify this, we trained a decoder to predict the timing of somatic action potentials based on the somatic subthreshold membrane potential, either with dendritic spikes and consequent spikelets (i.e., using the full biophysical model, v_soma_) or without dendritic spikes (by blocking dendritic Na^+^ channels, v_noNa_; STAR Methods). We found that action potential timings were substantially better predicted with dendritic spikes than without them as measured by precision and recall (Figure 2F) or a coincidence factor^46^ (Figure 2G). Measuring prediction performance using a receiver operating characteristic-based approach, as used in earlier studies,^13,47^ was not able to distinguish between these two cases, as this measure is prone to be dominated by the overwhelming number of time bins in which no spikes occur (Figure S2).

### Unitary architectures fail to accurately capture spikelets

To mathematically characterize the input-output transformation of the CA1 pyramidal cell, we optimized a cascade of subunits to predict the subthreshold somatic membrane potential at a given time, v_soma_, from the spike trains received by the cell up to that point (Figure 1B, see also Figure S3 and STAR Methods). Standard approaches to using such cascades employ static subunits whose output depends only on their instantaneous inputs or a simple linearly filtered version of their inputs.^9,11,13^ To increase the expressivity of our cascades, we extended previous approaches by allowing subunits to have internal dynamics, the parameters of which we could fit together with all the other parameters of the cascade (synaptic and subunit weights). Importantly, all these architectures remained unitary. In contrast to recent approaches deploying densely connected DNN-like architectures^13^ (Figure 1D), we restricted our analyses to simple, sparsely connected cascades, such that the number of pathways of information processing within the cascade remained minimal and, thus, the resulting architecture interpretable.

**Figure 3:**
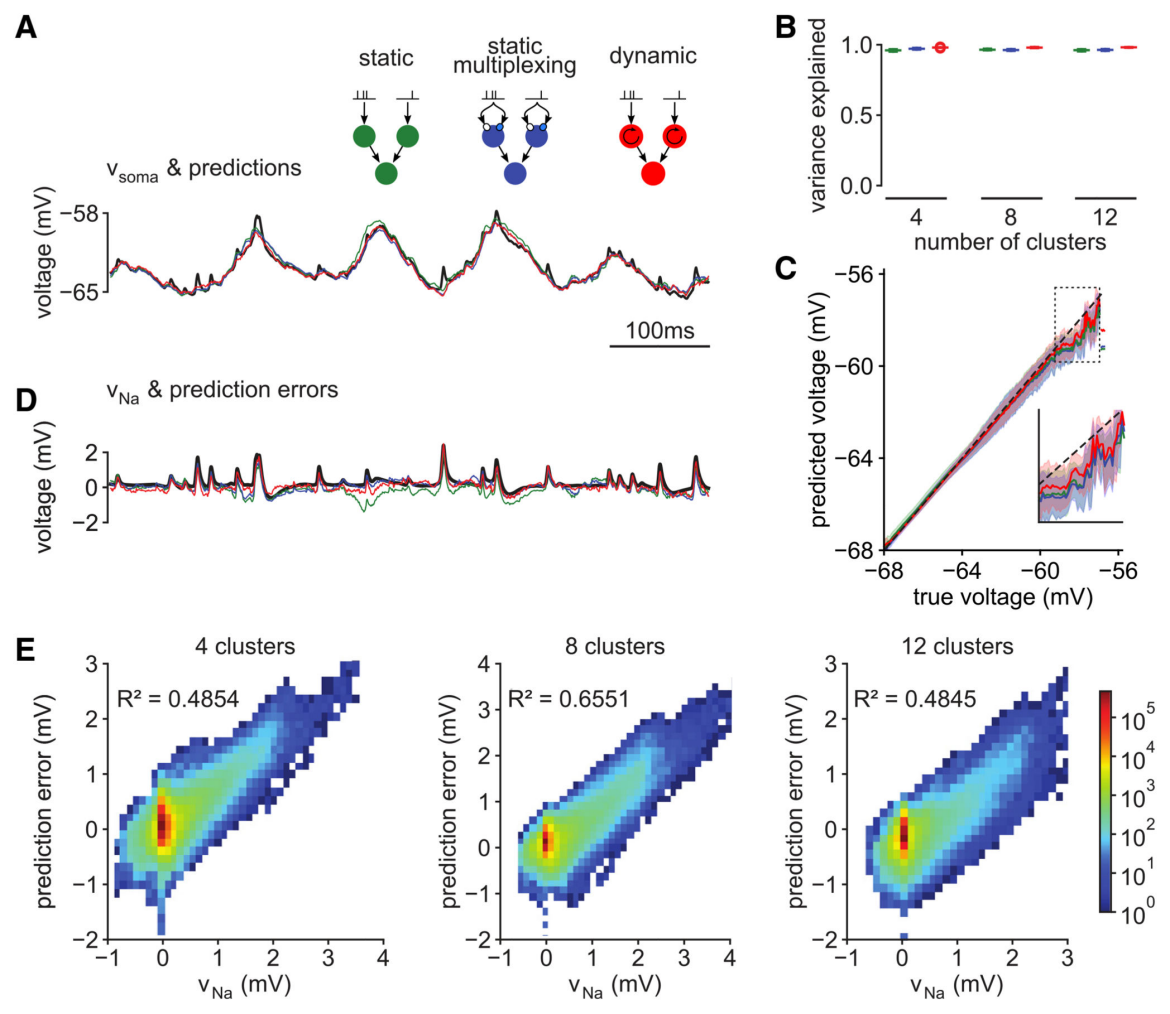
Unitary architectures fail to accurately capture spikelets (A) Sample somatic membrane potential trace of the biophysical model neuron (black) with predicted voltages from the static (green), static with multiplexing (blue), and dynamic (red) cascade models. Inset shows schematics of the architectures used: number of leaf subunits was 1 (receiving somatic inhibition) + 5 (number of main dendrites, for nonclustered synapses) + 4, 8, or 12 (number of synaptic clusters), with synapses assigned to the subunit corresponding to the clustering and location of that synapse in the biophysical model (see also STAR Methods). (B) Cross-validated performance (variance explained) of the three cascade types (colors as in A) for the three cluster arrangements (x axis, see also Figure 2B). (C) Predicted voltage as a function of the true voltage for the three cascade types (colors as in A). Inset shows enlarged view of the nearthreshold regime. (D) Sample traces of the Na^+^ differential voltage (v_Na_, black) and prediction error residuals of the three cascade types (colors as in A) during the same trial as shown in (A). (E) Pearson correlation (R^2^) and joint histogram of the dynamic cascade model’s prediction errors and the Na^+^ differential voltage (v_Na_) for the three cluster arrangements. Boxplots in (B) show median (horizontal line), 25th and 75th percentiles (box), ±1.5 interquartile ranges (whiskers), and outliers (circles) across 20 test trials. Lines and shaded areas in (C) show mean ±1 SD across 20 test trials. Data in (A), (C), and (D) are from the four-cluster arrangement. See also Figures S3–S5.

In line with earlier results,^9^ we found that a simple architecture with only two layers of static subunits explained 96% of the variance of the subthreshold somatic membrane potential (Figures 3A and 3B, green). This performance was on par with that of more complex models using deeper cascades^9^ and even more flexible, densely connected DNN architectures^13^ (see also Figure S4A). We also considered multiplexing, such that each input spike train contributed to two different nonlinearities within the same subunit (Figure 1C), which has been shown to improve performance at least as much as increasing the depth of the cascade.^9^ We found little to no increase in performance with multiplexing (96%–97%; Figures 3A and 3B, blue). As a final improvement, we replaced the static subunits of the cascade with dynamic subunits, which also achieved only a small improvement in performance (98%; Figures 3A and 3B, red).

Although these unitary models could predict the somatic response with high accuracy on average, a closer examination revealed systematic errors in these predictions. Specifically, we found that higher somatic membrane potential values (i.e., those nearer the firing threshold) were predicted more poorly, with a larger (downward) bias and a larger variance, and that this problem was present for all three models we considered so far (Figure 3C). These larger prediction errors seemed to stem from an inability of these models to correctly predict spikelets (Figure 3A, compare black with green, blue, and red).

To understand what causes the poor predictability of spikelets, we extracted the contribution of dendritic Na^+^ channels to the somatic membrane potential, v_Na_ (i.e., putative spikelets). We defined vNa as the difference between the somatic membrane potentials we recorded in the biophysical model with (v_soma_) and without the inclusion of dendritic Na^+^ channels (v_noNa_; see also Figure 2E, top):

(Equation 1) $${\text{V}}_{\text{Na}}={\text{V}}_{\text{soma}}-{\text{V}}_{\text{noNa}}.$$

Thus, v_Na_ included the direct contributions of dendritic Na^+^ channels to the somatic membrane potential (i.e., the additional depolarization their currents caused), as well as any indirect contributions they might have had (i.e., any interactions with other channels, e.g., NMDARs, whereby the additional depolarization caused by Na^+^ channels increased, or decreased, the currents through these other channels). However, these indirect contributions were minimal in our biophysical model (NMDAR currents barely changed when Na^+^ channels were blocked; Figures S1F and S1G). Therefore, virtually all contributions of NMDARs to the somatic membrane potential were represented in v_noNa_.

We observed that moment-by-moment fluctuations in v_Na_ were closely followed by fluctuations in the prediction error of the cascade models (Figure 3D) such that they were strongly correlated (Figure 3E). This remained the case even when using an entirely different, DNN-based class of unitary architectures (Figures S5A–S5C). These results suggested that it was the specific contribution of dendritic Na^+^ channels to v_soma_ that unitary architectures failed to capture accurately.

### Differences between V_noNa_ and V_Na_ architectures: Static versus dynamic subunits

**Figure 4:**
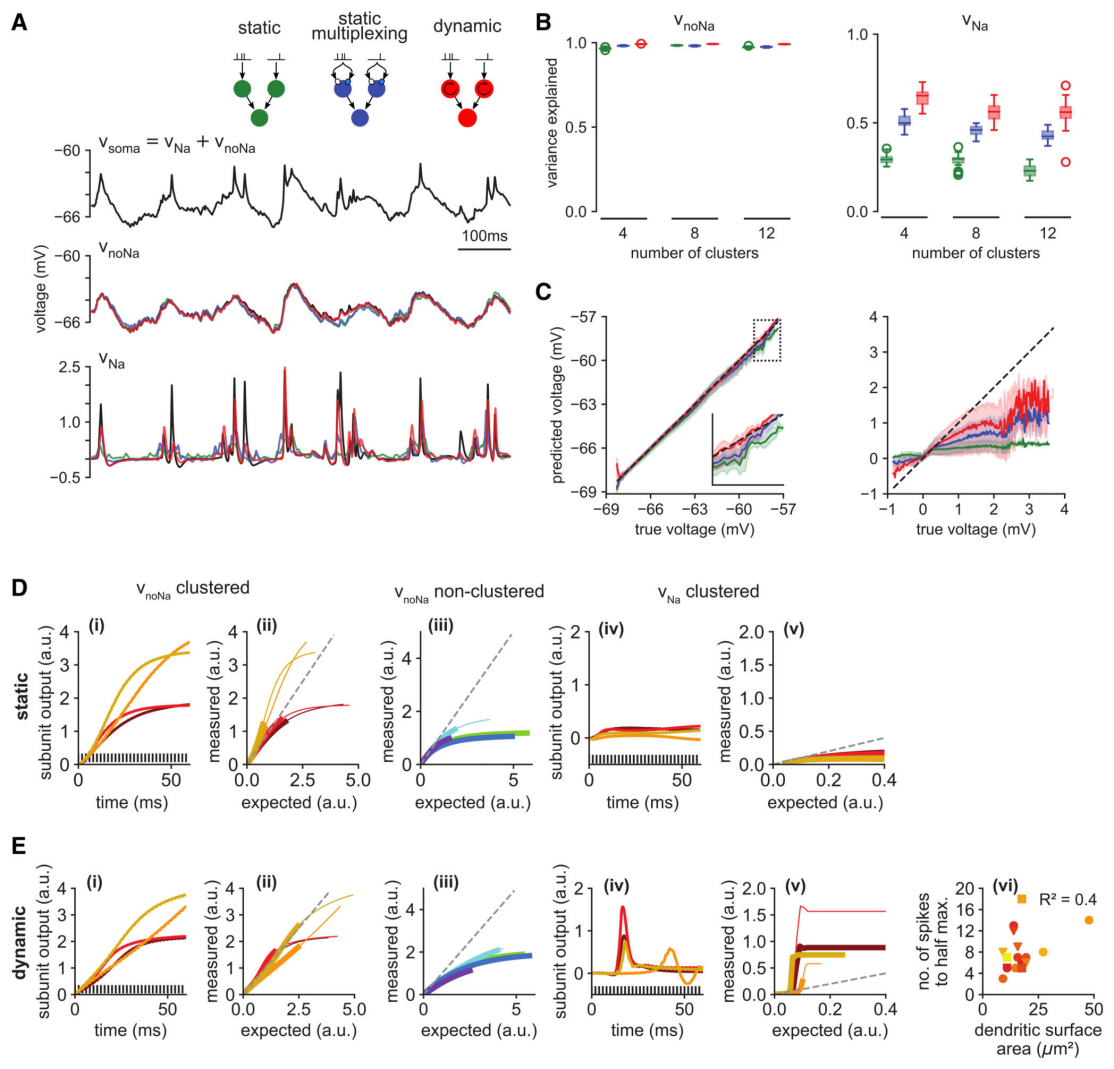
Differences between v_noNa_ and v_Na_ architectures: Static versus dynamic subunits (A) Sample traces of v_soma_ (black, top), v⊓oNa (middle), and v_Na_ voltages (bottom) with predicted voltages from the static (green), static with multiplexing (blue), and dynamic (red) cascade models (see also inset, same as in Figure 3A). (B) Cross-validated performance (variance explained) of the three cascade types (colors as in A) for the V_noNa_ (left) and v_Na_ voltages (right) in the three cluster arrangements (x axis, see also Figure 2B). (C) Predicted voltages as a function of the measured V_noNa_ (left, inset shows enlarged view of the near-threshold regime) and v_Na_ voltages (right) for the three cascade types (colors as in A). (D and E) Input integration in static (D) and dynamic (E) subunits fitted to the v_noNa_ (i–iii) or v_Na_ voltage response (iv–vi). (i) and (iv) show the responses of the subunits (colors) receiving clustered inputs to 30 spikes at 2-ms intervals. (ii) and (v) show the measured versus expected amplitudes of responses (such as those in i and iv, respectively), while varying the number of input spikes between 1 and 30. Expected response amplitude is the linear summation of responses to individual stimuli. Thick lines indicate the subunit response range during *in vivo*-like stimulation (Figure 2A). (iii) is same as (ii) for subunits receiving nonclustered (background) inputs. (vi) shows the threshold of each subunit nonlinearity (measured as the number of spikes required to reach half-maximal response) of the cascade model against the surface area of the corresponding dendritic branch receiving the clustered inputs in the biophysical model. Different symbols indicate fits with different cluster arrangements (only subunits generating dendritic Na^+^ spikes are included). Boxplots in (B) show median (horizontal line), 25th and 75th percentiles (box), ±1.5 interquartile ranges (whiskers), and outliers (circles) across 20 test trials. Lines and shaded areas in (C) show mean ±1 SD across 20 test trials. Data in (A), (C), (D), and (E) (i–v) are from the four-cluster arrangement. See also Figures S6–S9.

As spikelets riding on top of other fluctuations in the somatic membrane potential proved specifically challenging to predict for the cascade models, we proceeded by subdividing the task of predicting the complete somatic membrane potential, v_soma_, into separately predicting its two additive components (Equation 1; Figure 4A, top): only the spikelets, v_Na_ (Figure 4A, bottom) and the “rest,” i.e., the somatic membrane potential with dendritic Na^+^ channels blocked, v⋂oNa (Figure 4A, middle). We then asked if there were any key differences between the functional architectures that best predicted each of v_Na_ and v_noNa_. Specifically, we asked if they differed in the nature of the elementary operations their subunits performed, the number and connectivity of their subunits, and the way synapses were organized onto those subunits.

We found that V_noNa_ could be predicted by all cascades with high accuracy (variance explained 96% or more), with only little, if any, improvement with more complex cascades (multiplexing or dynamic subunits; Figures 4A, middle, and 4B and 4C, left; see also Figure S4B). In contrast, predicting v_Na_ was more challenging (variance explained less than 70%, even with cascades with >2 layers; Figure S4C). In this case, dynamic subunits more than doubled performance over static subunits (Figures 4A, bottom, and 4B and 4C, right, red versus green), while multiplexing achieved only more moderate improvements (Figures 4A, bottom, and 4B and 4C, right, blue versus green; similar results were obtained also with 3-fold multiplexing; Figure S4C). This remained the case even when static subunits were used in a densely connected DNN-based architecture, rather than a sparsely connected cascade (Figures S4C and S5D and S5E).

To understand why dynamic subunits were necessary for fitting v_Na_, but not V_noNa_, we examined how they processed their inputs in comparison to static subunits (Figures 4D and 4E). We found that fitting v_noNa_ required only the generation of slowly evolving (on the timescale of tens of milliseconds) responses, even for strong, clustered inputs (Figure 4E, i), with only a mildly superlinear integration of inputs (Figure 4E, ii), which even became sublinear for nonclustered inputs (Figure 4E, iii). This behavior could be expressed by static subunits as much as by dynamic subunits (Figure 4D, i-iii). However, dynamic subunits fitted to v_Na_ produced large but transient (spike-like) responses for strong stimuli (Figure 4E, iv) that were characterized by strong, step-like superlinearities (Figure 4E, v). Static subunits were unable to respond in this way (Figure 4D, iv and v) because of their fundamental inability to produce transient responses to sustained stimulation: once inputs reach threshold, a large response is necessarily maintained for the duration of the input. We also found that the step-like nonlinearity of the fitted dynamic subunits reflected a meaningful biophysical property of the corresponding dendritic branch: their threshold correlated with the membrane area of the dendritic branch receiving the clustered inputs (Figure 4E, vi), which is a major factor determining local input resistance.^48^

### Differences between v_noNa_ and v_Na_ architectures: Single versus multiple subunits

**Figure 5:**
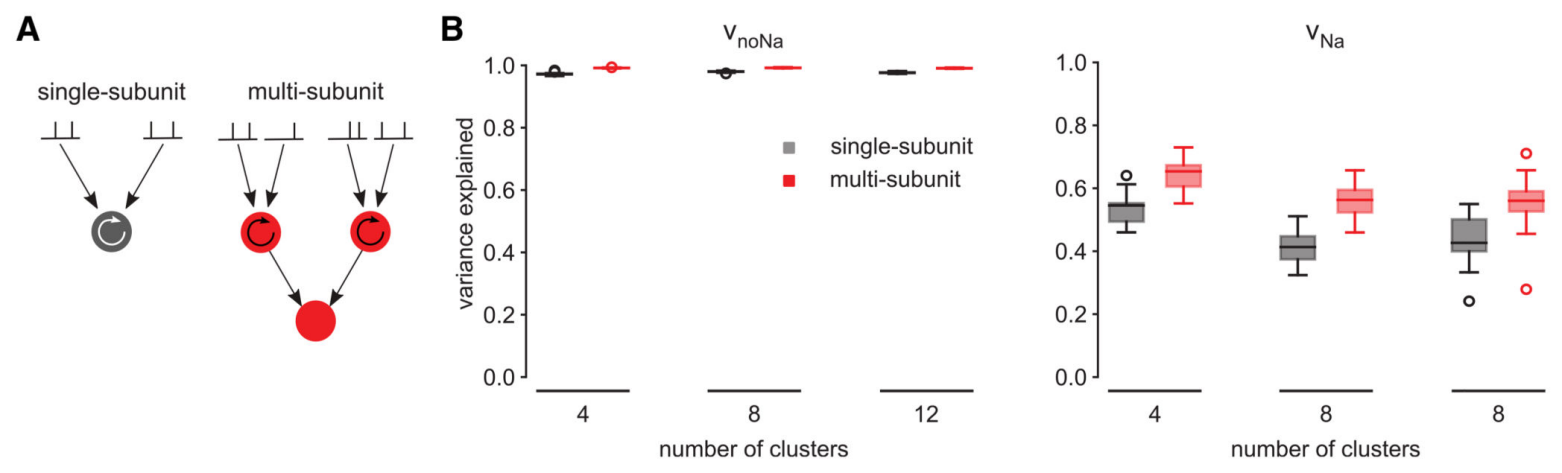
Differences between V_noNa_ and v_Na_ architectures: Single versus multiple subunits (A) Schematics of single- and multiple-subunit architectures compared in (B). (B) Cross-validated performance (variance explained) of the two architectures (colors as in A) for the v⋂oNa (left) and v_Na_ voltages (right) in the three cluster arrangements (x axis, see also Figure 2B). Boxplots in (B) show median (horizontal line), 25th and 75th percentiles (box), ±1.5 interquartile ranges (whiskers), and outliers (circles) across 20 test trials.

We next asked whether, other than their differences in the temporal processing of inputs, there were also differences in the spatial integration of inputs between v_noNa_ and v_Na_. (For a fair comparison, based on the results above, Figures 3, 4, and S4, we used dynamic subunits for both v_noNa_ and v_Na_.) Specifically, we investigated how much v_noNa_ and v_Na_ reflected genuinely multiple functional subunits. For this, for each of v_noNa_ or v_Na_, we compared the predictions of two cascades, one with a single subunit, and consequently with all synapses assigned to that subunit, and another one with multiple subunits, whose number scaled with the number of synaptic clusters in the biophysical simulations and with the “correct” assignment of synapses to those subunits that reflected the clustering of synapses in the biophysical simulations (Figure 5A; STAR Methods).

In line with previous studies,^9^ we found that the single-subunit model could predict v_noNa_ with an accuracy (within ~2% performance difference) similar to that of the multi-subunit model (Figure 5B, left). Having multiple subunits was more relevant for predicting v_Na_ (performance improved by ~30% from single-subunit models; Figure 5B, right). Although the improvement in prediction performance with multiple subunits for v_Na_ may still seem modest, performance could not be increased even using more complex architectures with more subunits (Figure S4C), suggesting that—at least within the model class we considered—the two-layer architecture with dynamic subunits was (locally) optimal.

### Robustness to model assumptions

To assess the robustness of our main findings, we introduced changes to the underlying biophysical model, and its inputs, to which we fit our cascade models. We first varied NMDA and Na^+^ conductances in ranges that still generated realistic responses in the model (Figures S1D and S1E). As in the original model, we found that v_Na_ strongly correlated with the prediction errors of a unitary architecture trying to predict v_soma_ (Figure S6A, cf. Figure 3E) and that v_Na_, but not v_noNa_, required multiple (versus single; Figures S6B and S6C, cf.Figure 5B) dynamic (versus static; Figures S6D and S6E, cf.Figure 4B) subunits to be predicted accurately.

Next, we modified the spatial clustering of inputs to the biophysical model so that, in line with recent findings,^49^ more local dendritic events (Na^+^ spikes and NMDA plateaus) were produced outside the place field of the cell, occasionally even in the absence of somatic action potentials (Figure S7). Our main results still remained unchanged under this modified input regime (Figure S8).

As our original approach involved fitting the cascade model to v_soma_ (Figures 3 and 4A), i.e., the somatic membrane potential with axosomatic action potentials blocked, our results so far ignored the potential effects of back-propagating action potentials (bAPs) on dendritic integration. Therefore, we simulated the biophysical model with axosomatic Na^+^ channels, and thus action potentials unblocked, and refit the cascade to its somatic membrane potential, vfull. As expected, in the biophysical model, the difference between vfull and vsoma was large during and immediately following an action potential (Figure S9A, top) due to the direct additional local depolarization of the soma. However, this difference was dominated by a stereotypical waveform that quickly (within ~25 ms) returned to near-zero levels after the action potential (Figure S9A, bottom). Similarly, while we found that bAPs were detectable in virtually all dendritic branches, their effects on the local membrane potentials also rapidly converged to zero in all but a subset of branches (Figure S9B, top). However, given the sparsity of somatic action potentials, even in these branches, bAPs contributed little overall to the local membrane potentials beyond an additive stereotypical waveform (Figure S9B, bottom). In line with this, when comparing the cascade models fit to v_full_ versus v_soma_, we found that input integration barely changed, as revealed by the near perfect correlation between the local subunit activations of either their v_noNa_ or their v_Na_ architectures (Figures S9C and S9D). These results suggest that the net effects of bAPs on dendritic integration were minimal, at least as seen in the soma.

### Differences between V_noNa_ and v_Na_ architectures: Synaptic organization

In the foregoing, we studied the internal properties of the v_noNa_ and v_Na_ architectures. We next asked how their external inputs, i.e., the synapses impinging the cell, are organized onto their subunits. For this, we developed a method to automatically discover the assignment of synapses to subunits (as well as their weights) in a data-driven way. This extended previous approaches^9,13^ used for our preceding analyses that only optimized synaptic weights using hand-picked synapse assignments.

**Figure 6:**
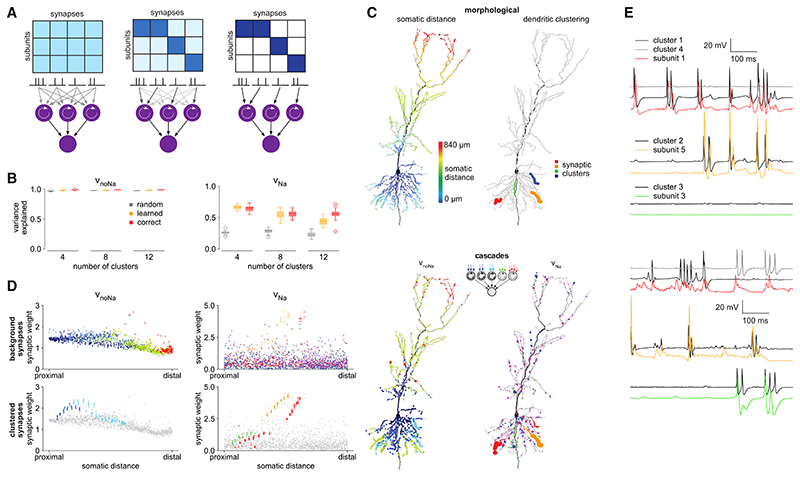
Differences between v_noNa_ and v_Na_ architectures: Synaptic organization (A) Schematic of the “annealed” optimization approach used for the automatic discovery of synaptic organization. (B) Cross-validated performance (variance explained) of a multi-subunit cascade (shown in A) with random (gray), optimized (orange), and hand-picked “correct” assignment of synapses to subunits in thev_noNa_ (left) and v_Na_ architectures (right) forthe three cluster arrangements (x axis, see also Figure 2B). Synaptic weights were always optimized for a given synapse assignment. (C) Synaptic organization in the biophysical model (top) and in the cascade model (bottom). Top: somatic distances of synapses (left) and locations of clustered synapses in the biophysical model (right, dot sizes are proportional to the frequency of dendritic Na^+^ spikes in the target branches). Bottom: optimized subunit assignments of synapses (dot colors, see also insets) and weights (dot sizes) in the v_noNa_ (left) and v_Na_ architecture (right). (D) Optimized weights of background (top) and clustered synapses (bottom) in the v_noNa_ (left) and v_Na_ architectures (right) against their somatic distances in the biophysical model. Colors reflect subunit assignment (as in C, bottom), and background synapses are shown in gray in the bottom plots for reference. (E) Sample local v_Na_ of the four clustered dendritic branches (black and gray) and subunit activations (color as in C, bottom right) for three subunits of the v_Na_architecture during two example time periods (top and bottom). Boxplots in (B) show median (horizontal line), 25th and 75th percentiles (box), ±1.5 interquartile ranges (whiskers), and outliers (circles) across five separate training datasets. Data in (C)—(E) are from the four-cluster arrangement. See also Figure S10.

We formalized the problem of discovering synaptic organization as a constrained optimization problem, which we solved by using an “annealing” approach. Specifically, the algorithm maximized the explained variance of the V_noNa_ or v_Na_ signal, such that it started from uniform synaptic weights and an all-to-all assignment of inputs to subunits and then gradually refined the model’s synaptic organization to achieve a sparse pattern, with each input being ultimately assigned to only one subunit with a non-zero synaptic weight (Figure 6A; STAR Methods). For a fair comparison, we used architectures with identical internal properties (multiple dynamic subunits) for both V_noNa_ and v_Na_ (but see also Figure S10 for v_noNa_ results with a single static subunit, which our previous analyses indicated to be more appropriate in that case).

We found that accuracy with the optimized synaptic assignments and weights was higher than with random assignments (but weights still optimized; Figure 6B, orange versus gray) and was comparable to that achieved with hand-crafted, “correctly” clustered synapse assignments (Figure 6B, orange versus red; optimized synaptic assignment models attaining over ~90% of the performance of hand-crafted models). The similar performances of the optimized and clustered assignments demonstrate the efficiency of our optimization approach and also validate our previous choices for clustered synapse assignments.

Next, we analyzed the optimized synapse organization for both v_noNa_ and v_Na_. As the biophysically defined weights (maximal conductances) of synapses were uniform in the biophysical model, any systematic differences between their effective weights in the optimized cascade models reflected functional differences between their contributions to the V_noNa_ or v_Na_ signals. For V_noNa_, synapse assignments mostly reflected the distances of the synapses from the soma, such that synapses in approximate “concentric rings” around the soma were assigned to the same functional subunit (Figure 6C, top and bottom left). Synaptic weights were broadly comparable for clustered and background (i.e., nonclustered) synapses. The weights of clustered synapses showed no apparent structure (Figure 6D, bottom left). The weights of background synapses were negatively correlated with somatic distance (Figure 6D, top left), reflecting the mostly nonregenerative nature of these signals. In contrast, for v_Na_, the assignment of synapses to functional subunits primarily reflected their clustering on the dendrite rather than their somatic distance (Figure 6C, top and bottom right), with background synapses having markedly lower weights than clustered synapses (Figure 6D, bottom right). Moreover, while background synaptic weights exhibited no obvious dependence on somatic distance (Figure 6D, top right), clustered synapses were positively correlated with it (Figure 6D, bottom right). This reflected the regenerative nature of v_Na_ signals and their dependence on the local membrane impedance, which is known to increase toward dendritic tips.^50,51^

Although our optimization objective considered only the explained variance of the somatic voltage response, we found that, with the synaptic organization discovered for v_Na_, even the local activations of the subunits closely resembled the local v_Na_ of the corresponding dendritic branches (Figure 6E). Occasional mismatches between the two (Figure 6E, top row) were simply due to the imperfect, sometimes many to one, correspondence between the dendritic clustering of synapses (Figure 6C, top right) and their assignment to functional subunits, as discovered by our optimization algorithm (Figure 6C, bottom right). This provided further evidence that the subunits of the v_Na_ architecture were biophysically interpretable.

**Figure 7:**
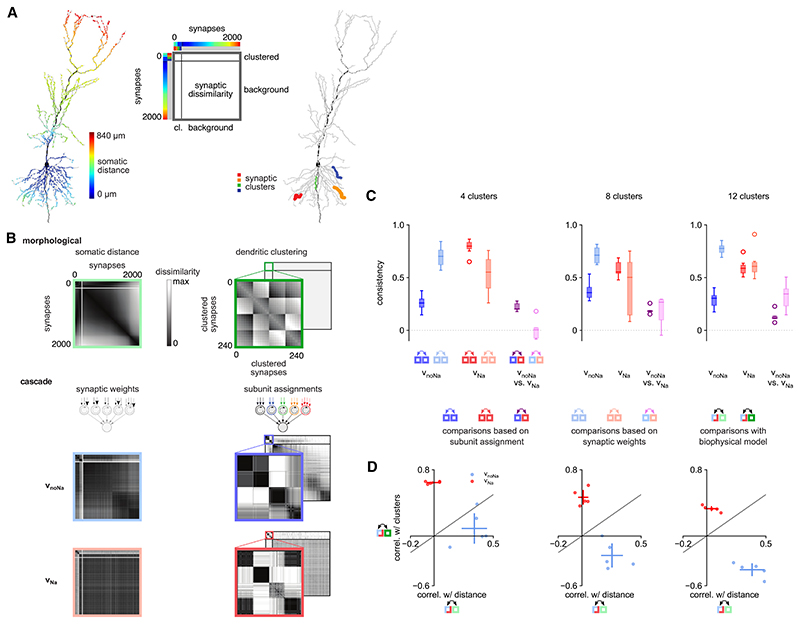
Functional orthogonality of v_nONa_ and v_Na_ architectures (A) Schematic for the construction of the synaptic dissimilarity matrices shown in (B). A synaptic dissimilarity matrix shows the dissimilarity between all possible pairs of synapses, ordered by their distance from the soma (outer color bars, color code is shown in left morphology, see also Figure 6C, top left), starting with the clustered synapses (inner color bars, color code is shown in right morphology, see also Figure 6C, top right). (B) Synaptic dissimilarity matrices based on the somatic distance (top left) and dendritic clustering (top right) of synapses, in the biophysical model, and the weight (bottom left) and subunit assignment (bottom right) of synapses in the cascade models trained to predict either v_noNa_ or v_Na_ (bottom two rows, averaged across fits to different training datasets). The dendritic clustering-based morphological dissimilarity matrix was computed only for clustered synapses and orthogonalized with respect to the corresponding (top left) block of the somatic distance-based morphological dissimilarity matrix (see also STAR Methods). Magnified top left blocks in the right column are shown to highlight characteristic block structure reflecting clustering. (C) Consistency of synaptic organizations within the V_noNa_ (blue) and v_Na_ (red) cascade architectures, as well as between them (purple), based on the subunit assignment (dark colors) or weights of their synapses (light colors, see also insets with colored boxes and arrows, and B for color code). Within- versus across-architecture consistency is measured as the Pearson correlation between dissimilarity matrices obtained by fitting training datasets of the same versus different kinds of voltage signals produced during different versus the same runs of the biophysical model. (D) Correlation (Pearson correlation coefficient) of the synaptic organizations for the v_noNa_ (light blue) and v_Na_ architectures (dark red) with the somatic distances (x axis) and dendritic clustering of synapses (y axis) for the three cluster arrangements. Each architecture is characterized by its more consistent synaptic dissimilarity matrix (cf. C): synaptic weight based for v_noNa_, and subunit assignment based for v_Na_. Clustering-based correlations (y axis) are computed only for clustered synapses (top left block of corresponding dissimilarity matrices, see also B). Boxplots in (C) show median (horizontal line), 25th and 75th percentiles (box), ±1.5 interquartile ranges (whiskers), and outliers (circles) across five separate training datasets (C, purple) or 10 training dataset pairs (C, blue and red). Crosshairs in (D) show mean ±1 SD along each axis across five training datasets. See also Table S1.

We also quantified the similarity between different optimized synaptic organizations, as well as their relationship to the organization of synapses in the biophysical model. For this, we employed a “representational similarity analysis”-based approach^52^and used the Pearson correlation between matrices measuring the (dis)similarity of pairs of synapses (Figure 7A; STAR Methods). For the cascade models, dissimilarities between synapses were defined based on one of the two defining aspects of their optimized organization. First, we used dissimilarities that depended solely (in a binary way) on whether two synapses were assigned to the same subunit (Figure 7B, middle and bottom right). In this case, no consistent synaptic organization could be found for v_noNa_across different training datasets (Figure 7C, dark blue). This was in line with our earlier finding that the v_noNa_ architecture can be effectively characterized by a single subunit (Figure 5B, left). In contrast, we found that the same synaptic organization was reliably discovered for v_Na_ across training datasets (Figure 7C, dark red). Critically, there was only minimal correlation between the synaptic organizations discovered for v_Na_ and v_noNa_, even for the same training set (Figure 6C, dark magenta).

Second, we used dissimilarities that measured (in a graded way) the absolute difference between the synaptic weights of a pair of synapses (Figure 7B, middle and bottom left). By this measure, synaptic organizations optimized for v_noNa_ were highly consistent and even more so than those optimized for v_Na_ (Figure 7C, light blue versus red), but the agreement across v_Na_ and v_noNa_ synaptic organizations was still low (Figure 7C, light magenta). These results indicated that, while the synaptic organization for v_noNa_ is most reliably defined by its synaptic weights, the v_Na_ synaptic organization is best defined by the cluster assignment of its synapses, and that there is very little in common between v_Na_ and v_noNa_ organization in general. (As the multi-subunit architecture was unnecessary and unreliably recovered for v_noNa_, we also repeated our analyses for v_noNa_ by fitting the synaptic weights in a single-subunit architecture and obtained very similar results; Figure S10.)

To better understand the factors contributing to the differences between the synaptic organizations of the V_noNa_ and v_Na_ architectures, we also created synapse-dissimilarity matrices based on the organization of synapses in the biophysical model. Specifically, we computed a somatic distance-based dissimilarity matrix, with each element measuring the absolute difference between the somatic distances (along the dendritic tree) of a pair of synapses (Figures 7A and 7B, top left) and a clustering-based dissimilarity matrix that depended on whether synapses belonged to the same dendritic cluster in the biophysical model (orthogonalized with respect to the distance-based matrix; Figures 7A and 7B, top right). We then compared each of these biophysical dissimilarity matrices with those derived from the cascade model as explained above. For each of the cascade models describing the V_noNa_ or v_Na_ architecture, we chose the dissimilarity matrix that most reliably characterized it (i.e., synaptic weight based for v_noNa_ and subunit assignment based for v_Na_; we also obtained similar results using the converse combination). In line with the qualitative patterns of synapse assignments observed in Figure 6C, we found a double dissociation in the relationship between how the synaptic organizations of the v_Na_ and V_noNa_ architectures were related to the dendritic clustering and the somatic distance of synapses (Figure 7D). Specifically, v_Na_ architectures were mostly correlated with the dendritic clustering of synapses and not with their somatic distances (Figure 7D, red), while v_noNa_ architectures were more strongly positively correlated with the somatic distances while not, or even negatively, correlated with the dendritic clustering of synapses (Figure 7D, blue).

Finally, we measured our ability to predict somatic action potentials with different functional architectures. For this we used the summed output of the v_noNa_ and v_Na_ architectures (i.e., architectures that were optimized for fitting the corresponding components of the subthreshold v_soma_). Prediction accuracy was slightly but significantly better than that achievable by using unitary (cascade, or DNN-based) architectures fitted directly to vsoma, and it was also on par with previously reported results using architectures that were explicitly optimized for action potential prediction^13^ (Figure S11).

## Discussion

Our results provide insights into dendritic integration in CA1 pyramidal cells (Table S1). In particular, they revealed a fundamental dichotomy in the way dendritic Na^+^ channels versus all other dendritic processes contribute to the somatic membrane potential. While the contributions of other processes, summarized in V_noNa_, are responsible for most of the subthreshold variability in the somatic membrane potential, the contribution of Na^+^ channels, represented by v_Na_, has a major influence on the timing of somatic action potentials. The functional architectures underlying these two contributions exhibit several key differences. In the V_noNa_ architecture, the entire dendritic tree functions as a single global subunit processing all synaptic inputs and expressing a static nonlinearity. This architecture is thus defined by the effective weights of synapses, which in turn mostly depend on their somatic distances. In contrast, in the v_Na_ architecture, the dendritic tree is best described by a cascade of multiple subunits, each of which corresponds to a small segment of the dendritic tree and processes only the corresponding subset of synaptic inputs. This architecture is mainly determined by how the synapses are assigned to different subunits, which in turn depends on the clustering of coactive synapses on the dendritic tree. The nonlinearity of subunits is dynamic and exhibits spiking-like behavior that is activated by strong and synchronous local inputs. Thus, synaptic inputs onto the same dendritic tree are processed by parallel functional architectures with distinct computational properties.

### Parallel versus unitary functional architectures

Ever since Rall’s pioneering work,^53^ most models of dendritic information processing, even when incorporating the effects of active dendritic conductances, considered the connectivity of their (biophysical or abstract) processing subunits to be fundamentally anchored to the morphology of the cell.^9,11,12,29,54–56^ This was a natural choice given the primary focus of many of these models on the spatial integrative properties of dendrites. However, as dendritic morphologies correspond to acyclic graphs, this meant that each synaptic input contributed to the output of the cell through a single pathway. Thus—by our definition—these models were necessarily unitary. Interestingly, even recent attempts that also tried to capture finer temporal aspects of dendritic integration,^9^ including DNN-based architectures that are otherwise detached from neuronal morphology,^13^ used an essentially unitary functional architecture. Therefore, our results showing parallel functional architectures within the same dendritic tree represent a major departure from previous abstractions of dendritic information processing.

Some previous studies hinted at the possibility of parallel architectures co-existing in the dendritic tree, albeit with somewhat ambiguous results.^1,29,57^ For example, while studies of dendritic integration using glutamate uncaging found different integration rules for dendritic Na^+^ versus NMDA spikes, the same studies also described substantial cooperativity between these forms of dendritic integration.^17,23,26,58^ This might seem to be incompatible with the parallel architectures we found. However, these studies used highly simplified input regimes *in vitro*, which do not necessarily generalize to the *in vivo* regime.^9^ Our approach, based on statistically rigorous analyses, allowed us to discover the existence of functionally parallel architectures underlying dendritic Na^+^-dependent and -independent signals under *in vivo*-like input conditions and to systematically characterize their differences.

Although the biophysical model that formed the basis of our investigations reproduced the cooperativity of Na^+^ and NMDA spikes^33^ (Figures S1A-S1E), the simple linear summation of the outputs of their corresponding parallel architectures suggests that this cooperativity does not play a major functional role during the integration of *in vivo*-like input patterns. Thus, these mechanisms may act more independently under *in vivo*-like conditions than previously thought based on *in vitro* evidence. Our finding that NMDA-mediated currents (under these conditions) barely change after blocking Na^+^ conductances in the biophysical model (Figures S1F and S1G) provides a simple biophysical explanation for this.

The parallel architectures we describe also differ from previous suggestions of dendritic multiplexing in critical ways. For example, hippocampal pyramidal cell dendrites have been demonstrated to switch between different modes of integration as a function of input synchrony,^17^ but this did not imply the co-existence of different modes of integration in parallel, at the same time. Neocortical pyramidal neurons have also been suggested to possess two parallel architectures,^59^ but such that these two architectures differ in the subset of synaptic inputs they process (basal versus apical) and the way their computations are reflected in the final output of the cell (spiking versus bursting), rather than in the actual computations they perform. The local multiplexing of synaptic inputs into different processing channels within the same subunit of a single functional architecture has been shown to account for a wide variety of phenomena,^9,60^ but previous work did not consider the parallel existence of entirely distinct functional architectures.

### Dynamic versus static computations in the dendritic tree

A key feature of the v_Na_ architecture we found was the dynamic nature of its subunits. Although the fundamental biophysical equations of cable theory describing signal propagation in passive dendritic trees are dynamic in nature,^53^ they result in a simple linear filtering of synaptic inputs and can thus be abstracted into a minimal functional architecture that convolves synaptic inputs with some fixed waveforms.^61^ This corresponds to our definition of a “static” subunit.

Recent work suggested that even the functional architectures of active dendritic trees can be described similarly, as long as the outputs of these convolutions are passed through static (pointwise) nonlinearities.^9,57^ This is also in line with what we found for the v_noNa_ architecture. Thus, our finding that the v_Na_ architecture specifically requires dynamic nonlinearities implies a qualitatively different mode of operation.

While dynamic nonlinearities are widely used to describe the time course of the somatic membrane potential in point-neuron models,^62^ they have rarely been incorporated into descriptions of dendritic functional architectures. When they were considered at all, previous work used simple forms of nonlinearities (e.g., feedback loops in generalized linear models or reduced two-compartmental biophysical models) and did not attempt to fit them to data, or fitted them only to spatiotemporally highly constrained input (and output) patterns.^43,60,63,64^ In contrast, we used gated recurrent units^65^ that allowed us to flexibly express a wide variety of dynamic nonlinear behaviors and to fit these systematically to richly structured *in vivo*-like inputs. Going forward, a fuller characterization of dendritic processing will likely require combining the flexibility of recurrent subunits, and their amenability to being fit to data, with the interpretability of low-dimensional dynamical systems.

### Sparse versus dense architectures

We used sparsely connected cascades for our analyses. These are less expressive than densely connected DNNs,^66^ which formally include sparse cascades as special cases. This means that sparse cascades can potentially have poorer predictive performance but, in return, they may require fewer training data and provide more interpretable results^67^ than DNNs whose computations can be notoriously difficult to interpret.^68^

For example, our key insights include the distinction between the V_noNa_ and the v_Na_ architectures in terms of using a single static versus multiple dynamic subunits and whether their synaptic organization is best characterized by their synaptic weights or subunit assignment (Table S1, see also above). Neither of these differences would have been straightforward to reveal with the densely connected DNNs used in previous work.^13^ First, the nodes of DNNs use purely static nonlinearities. Second, for the *in vivo*-like inputs we studied here, DNN nodes cannot be corresponded to functional subunits with individually identifiable contributions to the output of the cell (unless in the hypothetical special case in which they express a sparse architecture). At the same time, for the cell-type and prediction tasks we considered, using densely connected neural networks did not bring benefits in terms of predictive performance compared with sparsely connected (dynamic) cascades (Figures S4, S5, and S11). Future work will need to examine whether cascade-based parallel architectures yield similar performance gains and functional insights when applied to (neocortical) cell types for which both DNNs^13^ and cascadebased unitary architectures^9^ have been shown to have high predictive performance.

### Limitations of the study

An obvious limitation of our study is that we used a detailed biophysical model of a neuron as “ground truth” to which we fit our cascade models, in lieu of experimental recordings of an actual neuron. While this approach has now become standard in the field,^9,11,13^ it is still important to note that its conclusions rest on a necessarily imperfect biophysical model and assumptions about its inputs. Reassuringly, we found that the main conclusions of our study were robust to variations of some key parameters (Figures S6 and S8).

The other main component of our approach, the cascade models, could also be extended in various ways. For example, we assumed that synapses assigned to the same subunit differed only in the magnitude and not the time course of their contributions to the output of the subunit (and thus, ultimately, of the cell). However, dendrites show integration gradients even within single branches, where the precise location^50^and temporal order^25^of inputs can modulate the properties of the response. Incorporating such gradients into cascade models may provide improved prediction accuracy.

Another potentially important factor our cascade models do not capture is the interactions between locally generated active events in different dendritic branches.^30,58^While this issue is moot for the V_noNa_ architecture, which effectively consists of a single subunit (Figure 5), our preliminary results indicate that allowing dynamical interactions between subunits may indeed improve predictions for the v_Na_ architecture (Figure S12). Incorporating such interactions into cascade models, while still maintaining their interpretability, is an interesting direction for future research.

### Experimental predictions

The main prediction of our work is that the generation and propagation of dendritic Na^+^ spikes obey different rules than the general integration of synaptic inputs. To test this prediction, both a substantial fraction of the synaptic inputs of hippocampal pyramidal cells and their somatic membrane potential response should be simultaneously recorded (at high temporal resolution) under *in vivo* conditions. The somatic membrane potential response should be separated into V_noNa_ and v_Na_ components, and the techniques that we developed here and tested on simulated data should be used to predict each of these components from its synaptic inputs as recorded experimentally. We expect that predicting these components will require fundamentally different architectures, just as we found in our simulated data (Table S1). Specifically, for predicting v_noNa_, simple, single-subunit (one-layered) architectures with static subunits will suffice that, thus, take into account only the overall level of synaptic input to the neuron. This would be in line with earlier work using cascade models,^9^ but in contrast to work using DNNs.^13^ Conversely, when v_Na_ needs to be predicted, we expect that multilayered architectures with dynamic subunits will be necessary to take into account the detailed spatiotemporal configurations of inputs. This would be more in line with DNN-based conceptualizations of dendritic integration^13^ (although with the important caveat that dynamic rather than static subunits will be needed), but contrasts with earlier work using simpler cascade models.^9^

There are two particularly challenging aspects of such experiments: the separation of v_Na_ and V_noNa_ components and the comprehensive monitoring of synaptic inputs. First, the most direct way to dissect v_Na_ and V_noNa_ would require the specific blocking of dendritic Na^+^ channels. As the subunit compositions of dendritic versus somatic (or axonal) Na^+^ channels are highly similar,^69^ such a selective blockade might not be feasible in CA1 pyramidal neurons. Thus, as a more feasible, albeit also more indirect, approach, all Na^+^ channels of the cell could be blocked and model predictions compared for subthreshold somatic membrane potential fluctuations (under channel blockade) and somatic action potential timing (without the blocking of channels). These could serve as proxies for testing the differences between v_noNa_ and v_Na_. Alternatively, the v_Na_ component can be associated with the somatic effect of localized dendritic Na^+^ spikes generated in basal or oblique branches that appear as small-amplitude spikelets. These spikelets can be detected (e.g., in the first derivative of the membrane voltage^23,70^) while injecting a small hyperpolarizing current to prevent somatic spiking.

Second, monitoring synaptic inputs with sufficiently high temporal precision will require advances in the temporal resolution of calcium and glutamate sensors. The most recent variants (iGluSnFR3 and GCaMP8) are able to resolve single action potentials by imaging neuronal somata or individual synaptic boutons *in vivo*.^71,72^ Glutamate sensors will be preferable over Ca^2+^ or voltage indicators, as they are not affected by bAPs. Ultimately, microscopic techniques for imaging these sensors at scale, at a large fraction of synaptic inputs, will also be necessary.^73,74^ Until such techniques become routinely available, more targeted monitoring of a smaller fraction of inputs may also be adequate, e.g., focusing on dendritic branches with clustered inputs, which our results suggest to be critical for predicting v_Na_. More generally, computational analyses can also be used to establish the minimal fraction of synaptic inputs that needs to be monitored to reliably estimate the functional architecture of a neuron.

### Functional implications

Whether cortical neurons operate in a fundamentally rate- or spike timing-based coding regime has been a contentious issue for decades.^75^ In particular, hippocampal pyramidal neurons under *in vivo* conditions behave as place cells and are known to employ in parallel both a rate- and a timing-based code.^76,77^ Our results suggest that the dominant contributions of the V_noNa_ and v_Na_ architectures are respectively to these two different aspects of the cell’s output and, conversely, that they are mostly driven by different aspects of a cell’s inputs (overall level and fine spatiotemporal pattern, respectively). Thus, our findings of two parallel functional architectures in the same dendritic tree may offer mechanistic insights into the genesis and control of the parallel rate and temporal coding of hippocampal place cells. These insights may also generalize to other brain areas where a similar multiplexing of rate and temporal codes has been observed.^78^ More generally, once such multiplexed single-cell computations are incorporated into formal models of neural circuit dynamics, they may change our understanding of circuitlevel computations. For example, they may explain phenomena at the level of single neurons that had been ascribed to circuitbased mechanisms.^79^

We found that the v_noNa_ and v_Na_ contributions to the somatic membrane potential also differed in the set of biophysical and morphological properties of the cell that mainly determined their underlying architectures. As different plasticity mechanisms operate on these cellular properties, this raises the possibility that they may also be able to specifically modify one or the other architecture. For example, classical forms of synaptic plasticity changing synaptic weights^6,80^ may mostly affect the v_noNa_ architecture. In contrast, structural forms of plasticity that determine the clustering of synapses^81^ may play a dominant role in modifying the v_Na_ architecture. Finally, perhaps the best candidate for an architecture-specific plasticity mechanism is the more recently described branch-strength potentiation that has been shown to specifically affect the propagation of dendritic Na^+^spikes.^23^ As such, branch-strength potentiation may be an ideal substrate for “setting the switches” in the v_Na_ architecture. Our approach allows the data-driven dissection of these two functional architectures and thus paves the way for studying their modification over time by the interplay of an assortment of ongoing plasticity mechanisms.

## Star*Methods

### Key Resources Table

**Table:** 

Reagent or Resource	Source	Identifier
Software and algorithms		
Code for cascade models, biophysical models, and simulated input synapse spike trains	Zenodo	https://zenodo.org/record/7677374
NEURON 7.6.2	NEURON	https://github.com/neuronsimulator/nrn
Python 3.7	Python	https://www.python.org
PyTorch 1.10.0	PyTorch	https://pytorch.org/

## Resource Availability

### Lead contact

Further information and requests for resources should be directed to and will be fulfilled by the lead contact, Young Joon Kim (yjkimnada@gmail.com).

### Materials availability

No reagents, materials, or chemicals were used or generated in this study.

### Data and code availability

- All original code has been deposited at Zenodo and is publicly available as of the date of publication (seekey resourcestable).
- All simulated data used for our analysis can be generated from the aforementioned deposited code. No other original data was used in this study.
- Any additional information required to reanalyze the data reported in this paper is available from thelead contactupon request.

## Experimental Model And Study Participant Details

No *in vitro* samples, *in vivo* models, or human participants were involved in this study.

## Method Details

### Biophysical model CA1 neuron

To generate target membrane potential responses, we simulated the CA1 neuron model from Jarsky et al.^22^ modified as in Ujfalussy et al.^33^ to better account for dendritic processing of synaptic inputs in CA1 pyramidal cells. Full details of the biophysical model and the generation of synaptic inputs can be found in Ujfalussy et al.^33^ In short, the model included AMPA and NMDA excitation and slow and fast GABAergic inhibition and voltage gated Na^+^, delayed rectifier K^+^ and A-type K^+^ channels with location-dependent densities and kinetics as described in Ujfalussy et al.^33^ We did not include dendritic Na^+^ hotspots^21^ in the current model, as their presence in hippocampal CA1 pyramidal cells has not been unequivocally established and because we found sufficiently rich dynamical behaviour, including the reliable generation of dendritic Na^+^ spikes, even without such hotspots. We simulated three different versions of the model: 1. as a target for predicting somatic action potentials (APs), a “full” version including both somatic and dendritic active conductances (v_full_; see Table S2 for the list of figures showing results using the output of this model, and modes of use); 2. as one of the targets for training cascade models (and to study somatic AP predictions), a version with the maximal conductance of somatic and axonal (but not dendritic) Na^+^ channels set to 0 (v_soma_; see Table S2); and 3. as an alternative target for training cascade models (and to study somatic AP predictions), a version with the maximal conductance of Na^+^ channels set to zero in all compartments (v_noNa_; see Table S2). In all other respects (passive properties, other active conductances, synaptic locations and inputs, etc.), all simulations of the biophysical model used the same set of parameters, except in Figures S1D and S1E and S6 where we varied the dendritic Na^+^and NMDA conductances to test the robustness of results. Finally, we also used differences between the outputs of these models for various analyses. For analysing spike predictions (see below), we defined v_AP_ as v_AP_ = v_full_-v_soma_ (Table S2). As yet another target for training cascade models, we defined v_Na_ as v_Na_ = v_soma_-v_noNa_ (see also Equation 1; Table S2).

We distributed *S*^e^ = 2000 excitatory and *S*^i^ = 200 inhibitory synapses throughout the dendritic tree of the CA1 model neuron to account for the majority of its inputs from local interneurons and CA3 neurons. Of the 2000 excitatory synapses, 240 of them were chosen for functional clustering on the postsynaptic dendritic tree into 4, 8, or 12 clusters (of 60, 30, or 20 synapses, each, respectively). The locations of the synaptic clusters were selected amongst the dendritic compartments with lengths longer than 60 μm and the distance between synapses within a cluster was set to 1 μm. The remaining excitatory inputs were distributed uniformly throughout the dendritic tree, except in Figures S1F and S1G, Figures S7 and S8, where 4 additional sets of 20 inputs were chosen for functional clustering (Figure S7B). The peak activity of these additional input clusters was outside the place field of the postsynaptic neuron (Figure S7A). The 200 inhibitory synapses were divided into two groups such that 80 of them targeted the soma and the apical trunk to simulate strong perisomatic inhibition, while the remaining 120 were distributed randomly throughout the dendritic tree.

### Input regime

The input spike trains to the CA1 cell (and to the cascade models, see below) were designed to mimic neural activity in the hippocampus of a rodent continuously running around a circular 2 m-long track. Excitatory synapses received the spike trains of abstract presynaptic CA3 place cells as they displayed a single, idealized place field, were modulated by the ongoing 8 Hz theta oscillation, and exhibited theta phase precession.^9^ Inhibitory inputs were weakly modulated by theta oscillation, had an average firing rate of 7.4 Hz and were spatially untuned.

Specifically, the input spike train from excitatory presynaptic cell *i*, *s_i_*(*t*), was generated by a stochastic GLM with inhomogeneous Poisson spiking (at time resolution Δ*t* = 1 ms):

(Equation 2) $$s_{i}(t)∼\text{Poisson}\;\left(\text{Δ}t\lambda_{i}(t)\right)$$

based on an intensity function, *λ*_*i*_(*t*), that was tuned to a set of external and internal covariates,^33,82,83^ and was defined as

(Equation 3) $$\lambda_{i}(t)=\exp\left(a_{i}(t)\right)+\bar{\lambda }$$

where

(Equation 4) $$a_{i}(t)=w_{\varphi }^{\left(i\right)}\varphi (x(t),\psi (t))+w_{\chi }^{\left(i\right)}\int_{0}^{t}\chi (t-\tau )s_{i}(\tau )\text{d}\tau$$

is called the activation, $\bar{\lambda }$ is the baseline firing rate, *φ*(*X*, *ψ*) denotes a set of basis functions tuned to spatial location, *x*, and theta phase, ψ, 𝓧(·) is a set of kernels filtering the presynaptic cell’s own output, and $W_{\varphi }^{\left(i\right)}$ and $w_{\chi }^{\left(i\right)}$ are the presynaptic cell’s coefficients corresponding to these two types of covariates. To model spatial tuning and phase precession, we used basis functions co-tuned to spatial location and theta phase. Specifically, we used *N_basis_* = *N_x_*·*N_ψ_* = 160 (factorised) Gaussian basis functions with *N_x_* = 40 spatial and *N_ψ_* = 4 temporal components uniformly tiling the space with standard deviations *σ_x_* = 5 cm and *σ_ψ_* = π/2 radians. The parameter ***w**_φ_*, defining a spatio-temporal tuning curve-template shared by all presynaptic cells, was fitted to the spike density vs. location and theta phase data of Skaggs et al.,^39^ with the diameter of the place field being *d* ≈ 30 cm. In order for presynaptic tuning curves to tile the space evenly, we then generated each individual $W_{\varphi }^{\left(i\right)}$ by permuting the parameters in w4 such that it expressed the same spatio-temporal tuning curve up to a random shift along the spatial dimension. The average firing rate of the presynaptic place cells was chosen randomly from a gamma distribution with shape and rate parameters *α* = 3 and *β* = 6 corresponding to a 0.5 Hz grand average presynaptic firing rate,^35^ after accounting for the low release probability of hippocampal synapses^84^ (*p_rel_* ≈ 0.2). To also account for the spatially untuned activity of non-active place cells (which we did not simulate explicitly), the parameter $\bar{\lambda }$ was set to 0.1 Hz, leading to a final average input rate of 0.6 Hz for each simulated input. The term $w_{\chi }^{\left(i\right)}\int_{0}^{t}\chi (t-\tau )s_{i}(\tau )d\tau$ captures the effect of past presynaptic spikes on the input rate^83^ and was included to model a short (~ 5 ms) refractory period in the inputs.

The simulated animal was moving at a constant 20 cm/s speed, and in each trial we simulated the target somatic membrane potential response *v_soma_*(*t*) for 10 s at *δt* = 0.2 ms resolution.Figure 2 illustrates an example trial with all the input synaptic events (Figure 2A) together with the biophysical model’s somatic membrane potential response (Figure 2D). We fitted our cascade models to 980 runs with different input spike patterns but identical firing rates and reported the performance on 20 held-out test trials. For the architecture dissimilarity analysis (Figure 7D and 7E), we generated 5 different datasets of 1000 trials with presynaptic place field properties independently resampled from their corresponding distributions.

### Cascade models

All cascade models are summarized by the following equations:

(Equation 5) $$\tilde{v}(t)=\sum_{n=1}^{N}C_{0n}y_{n}(t)$$

(Equation 6) $$y_{n}(t)=F_{n}\left(s_{n}^{\text{e}}(0\ldots t),s_{n}^{\text{i}}(0\ldots t),{\bar{y}}_{n}(0\ldots t)\right)$$

(Equation 7) $$s_{n}^{\text{e}/i}(t)=C_{n}^{\text{syn,e/i}\;}\cdot \left(w^{\text{syn},\text{e}/\text{i}}⊙s^{\text{e}/\text{i}}(t)\right)$$

(Equation 8) $${\bar{y}}_{n}(t)=\sum_{m=1}^{N}C_{nm}y_{m}(0\ldots t)$$

where $\tilde{v}(t)$ is the final output (root subunit) of the cascade, *y_n_*(*t*) is the output of leaf subunit *n* (out of *N*) at time *t*, **C** is a binary matrix defining the connectivity between the subunits (a fully connected tree, with notional node 0, the root of the tree, corresponding to the final output), *F_n_*(·, ·, ·) represents the transformation carried out by a single model subunit (see below) on the history of its weighted excitatory / inhibitory input spike trains, $s_{n}^{\text{e}/\text{i}}(0\ldots t)$, and of its inputs from other subunits, ${\bar{y}}_{n}(0\ldots t),C_{n}^{\text{syn},\text{e}/\text{i}}$ is the corresponding row of the synapse-to-subunit assignment matrix C^*syn,e/i*^ (of which the columns, corresponding to synapses, are constrained to sum to one, and which has binary elements except while optimisation is still ongoing as shown in Figure 6), **w**^syn,e/i^ is a vector of scalar synaptic weights (with dimensionality equal to the total number of excitatory / inhibitory synapses, *S*^e^ and *S*^i^), **s**^e/i^(*t*) collects the spike trains arriving at all the excitatory / inhibitory synapses, and ⊙ denotes element-wise multiplication. Out of the parameters, **C** is fixed by the architecture, **C**^syn,e/i^ is either fixed or optimized, and **w**^syn,e/i^ is always optimized (constrained to be non-negative, see below).

We used different model architectures that differed in the number (*N*) and connectivity of their subunits (**C**), and their synapse-to-subunit assignment matrix (**C**^syne/i^). The two-layer architectures shown in the main text (Figures 3, 4, 5 and 7, see also Figures S4, S10, and S11) consisted of a number of leaf subunits, all feeding into the final output (root subunit) of the model (Equation 5). There was a separate subunit dedicated for each synaptic cluster (4, 8, or 12), 5 separate leaf subunits dedicated to non-clustered inputs targeting the 5 main dendritic branches of the neuron, and an additional subunit receiving the inhibitory input arriving at the soma of the biophysical model (Figure S3, see also below).

In some of our figures, we used a different number of leaf subunits. The “single subunit” model in Figures 5, S6B, S8B, S10, and in Figure S4 (“global”) only had a single leaf subunit. In the simulations where we optimized the synaptic organization (Figures 6 and 7), we used as many leaf subunits as the number of input clusters plus one (but note that in this case the actual synapse assignments used for initializing the optimization process did not reflect the correct assignment of synapses to subunits). The 3-layer architectures in Figure S4 used 2 subunits for each synapse cluster (see below). In simulations with altered Na^+^ or NMDA conductances (Figure S6), all background excitatory and inhibitory inputs targeted a single leaf subunit. Architectures fit to simulations with out-of-place field clusters (Figure S8) did not use separate subunits for the out-of-place field input clusters, and all inputs apart from the main cluster (i.e., background and out-of-place field clusters) were targeting a single leaf subunit. Finally, in the simulations studying the interactions between dendritic branches (Figure S12) we only used a single subunit that received the same input spike trains as a two-layer cascade model with separate subunits (see below).

The synapse-to-subunit assignment matrix, **C**^syn,e/i^, was either optimized (Figures 6 and 7, see below), or set by hand (to the “correct”assignment). In the latter case, for multi-subunit models, we used a separate leaf subunit to process the excitatory inputs arriving from each synaptic cluster along with those that synapsed onto the same dendritic branch of the biophysical model, and 5 subunits for non-clustered synapses, each receiving inputs from the background synapses targeting one of the 5 main dendritic subtrees of the cell branching from the soma (Figure S3B). The inhibitory subunits, which were spread uniformly across the dendritic tree of the biophysical model were also assigned to their respective subunits based on their morphological locations except for inhibitory synapses targeting the soma, which were placed on a separate nonlinear subunit. The outputs of all leaf subunits were integrated by a single, linear root subunit serving as the output of the cascade model.

The three-layer architectures in Figure S4 were constructed by starting from the 2-layer architectures (see above), and inserting an intermediate subunit between the root subunit and each leaf subunit that corresponded to a synaptic cluster. All input synapses that lay on the dendritic path between the soma and the clusters in the biophysical model were assigned to this intermediate subunit, rather than the cluster subunit. If a synapse in the biophysical model lay on the paths for several clusters (because the clusters were placed on sister branches), then the corresponding elements of **s**(*t*) and **w**^syn,e/i^ (and column of **C**^syn,e/i^) were duplicated in the cascade model. This way, synapses corresponding to the same biophysical synapse could target multiple intermediate subunits while being associated with unique weights in each.

Besides their architectures, we also varied the types of subunits comprising the cascade models. For this, we used two fundamentally different model classes that differed in the transformations their subunits implemented (*F_n_*(·, ·, ·) in Equation 6). First, following the hierarchical linear-nonlinear models of Ujfalussy et al.,^9^ the “static” model class used a classical linear-nonlinear transformation:

(Equation 9) $$F_{n}\left(s_{n}^{\text{e}}(0\ldots t),{\text{s}}_{n}^{\text{i}}(0\ldots t),{\bar{y}}_{n}(0\ldots t)\right)=\sum_{j=1}^{N_{\text{ch}}}w_{nj}\tanh\left({\bar{y}}_{n}(t)+x_{nj}^{\text{e}}(t)+x_{nj}^{\text{i}}(t)+b_{nj}\right)$$

where *j* indexes the input channels of the subunit (*N*_ch_ = 1 for non-multiplexed, or “1-plex” models, and *N*_ch_ = 2 for the multiplexed models of the main text, while *N*_ch_ = 3 was also used for Figure S4), *w_nj_* is the output weight of channel *j* of subunit *n*, *b_ni_* is a constant baseline input, and $x_{nj}^{\text{e}/\text{i}}$ is the temporally filtered excitatory and inhibitory input spike trains, $s_{n}^{\text{e}/\text{i}}$, respectively:

(Equation 10) $$x_{nj}^{\text{e}/\text{i}}(t)=\int_{0}^{\infty }s_{n}^{\text{e}/\text{i}}(t-\tau )\kappa_{nj}^{\text{e}/\text{i}}(\tau )\text{d}\tau$$

with the filtering kernels parameterized through a set of *K* = 30 raised cosine bases functions^83^ (shared across subunits, channel, and excitatory/inhibitory inputs):

(Equation 11) $$\kappa_{nj}^{\text{e}/\text{i}}(\tau )=\frac{1}{2}\sum_{k=1}^{K}\alpha_{njk}^{\text{e}/\text{i}}g\left({\bar{\tau }}_{k}(\tau )\right)\left[\cos\left({\bar{\tau }}_{k}(\tau )\right)+1\right]$$

where the subunit- and channel-specific $\alpha_{njk}^{\text{e}/\text{i}}$ coefficients were optimized (one set for each of excitatory and inhibitory kernels, see below), and $\bar{\tau }(\tau )$ is “rescaled” time

(Equation 12) $${\bar{\tau }}_{k}(\tau )=a_{k}\log\left(\tau +c_{k}\right)-\varphi_{k}$$

with fixed (i.e. not optimized) constants *a_k_* = 30 and *c_k_* = 1 for all *k*, *φ_k_* ranging uniformly from 0 to 14.5 *π*, and $g(\bar{\tau })$ simply ensuring that only the first period of each cosine bump contributes to the kernel (i.e. $g(\bar{\tau })=1$ when $\bar{\tau }\in [-\pi ,+\pi ]$ and $g(\bar{\tau })=0$ 0 otherwise).

Finally, for the dynamic models, we modeled each subunit as a standard gated recurrent unit (GRU).^65^ The internal dynamics of a GRU subunit were defined as:

(Equation 13) $$x_{n}(t)={\bar{y}}_{n}(t)+s_{n}^{e}(t)-s_{n}^{i}(t)$$

(Equation 14) $$r_{n}(t)=sig\left(w_{n}^{\text{ir}}x_{n}(t)+W_{n}^{\text{hr}}h_{n}(t-\delta t)+b_{n}^{r}\right)$$

(Equation 15) $$z_{n}(t)=sig\left(w_{n}^{\text{iz}}x_{n}(t)+W_{n}^{\text{hz}}h_{n}(t-\delta t)+b_{n}^{z}\right)$$

(Equation 16) $$k_{n}(t)=\tanh\left(w_{n}^{\text{ik}}x_{n}(t)+b_{n}^{k}+W_{n}^{\text{hk}}\left(r_{n}(t)⊙h_{n}(t-\delta t)\right)\right)$$

(Equation 17) $$h_{n}(t)=\left(1-z_{n}(t)\right)⊙k_{n}(t)+z_{n}(t)⊙h_{n}(t-\delta t)$$

with the output of the subunit defined as:

(Equation 18) $$F_{n}\left(s_{n}^{e}(0\ldots t),s_{n}^{i}(0\ldots t),{\bar{y}}_{n}(0\ldots t)\right)=\sum_{j=1}^{G}w_{nj}^{h}h_{nj}(t)+b_{n}$$

with *x_n_*(*t*) representing the synaptic input into the *n*th GRU subunit, ${\bar{y}}_{n}(t)$ denoting the inputs from other subunits (as in Equation 8), **sig**(·) and **tanh**(·) representing element-wise applications of the logistic sigmoid and hyperbolic tangent functions to their respective inputs, and **1** being a vector of 1’s, and *G* being the hidden dimensionality of the GRU (*G* = 20, unless otherwise stated).

To allow for nonlinear dynamic interactions both within and between subunits, we trained a dynamic casacde model with dendritic interactions (Figure S12, purple). This casacde model received input synaptic spike trains, $s_{n}^{e/i}(t)$, for every subunit and processed them all together within a single, global dynamic GRU subunit (with *G =* 100) to output a final somatic membrane potential prediction.

All models were trained via PyTorch^85^ with the Adam optimizer.^86^ For fitting v_soma_, v_noNa_, and v_Na_, we used the mean squared error between the predicted and true somatic voltage as the loss function and a learning rate of 0.005. A batch size of 5 trials was used throughout training. The parameters of the dynamic cascades were initialized with standard normal random initialization while the parameters of the static models were initialized by randomly sampling them from a normal distribution with *s* = 0.01.

For training while incorporating back-propagating action potentials (bAPs) we adopted a two stage procedure. First, cascade models were trained to separately predict v_Na_ and v_noNa_ as explained above. Second, a GRU decoder was trained to predict v_AP_ from the sum of the v_Na_ and v_noNa_ predictions, also as above (i.e. minimizing mean squared error). Finally, the cascade models and the GRU decoder were jointly trained to predict v_full_ via the sum of the v_noNa_, v_Na_, and v_AP_ predictions (Figures S9C and S9D; again minimizing mean squared error).

For spike train prediction (Figure S2, S11A and S11B), we used a GRU-based decoder, of which the output represented the predicted instantaneous spiking probability, and trained it using cross-entropy loss.

Critically, all spike prediction metrics and variance explained values reported in the paper were computed using a cross-validation approach, measuring mean squared error on a held out test data set (see above). This protects against overfitting and avoids unduly preferring overflexible models in our comparisons.

### Automatic discovery of synaptic organization

To discover the optimal synaptic organization in Figure 6 besides the standard parameters of the dynamic model (the weights, **w**, bias, **b**, and the parameters in Equations 13–18), we also optimised the *N×S* binary synaptic connectivity matrix **C**^syn,e/i^, where *S* = *S*^e^ + *S*^i^ is the total number of synapses. Each excitatory and inhibitory synapse was optimised independently so that the gradual assignment of one synapse to its respective subunit did not influence the assignments of the others. We started the optimisation from a full connectivity model where each input was connected with each subunit and optimised the elements of the matrix to minimise mean squared error between the predicted and true the true somatic membrane potential by the model as described above.

Throughout optimization, we ensured that the columns of **C**^syn,e/i^ sum to one by using the following parameterization:

(Equation 19) $$C_{nl}^{\text{syn},\text{e}/\text{i}}\left(\omega^{\text{e}/\text{i}},\beta \right)=\frac{\exp\left(\beta \omega_{nl}^{\text{e}/\text{i}}\right)}{\sum_{n^{\prime }}\exp\left(\beta \omega_{n^{\prime }l}^{\text{e}/\text{i}}\right)}$$

where $\omega_{nl}^{\text{e}/\text{i}}$ is the connection parameter between input *l* and subunit *n*. The β parameter was the inverse temperature hyperparameter and it allowed us to gradually enforce sparse connectivity by increasing it over iterations θof the optimization procedure,^87,88^ *β* = *β*(*θ*). Specifically, the inverse temperature started at *β*(0) =1 and was increased exponentially over iterations:

(Equation 20) $$\beta (\theta )=e^{-\theta /\tau }$$

with **τ** = 724 steps. This way the hyperparameter reached β(5000) ≈1000, at which it was no longer increased any further. At test time, we replaced the tempered softmax with the hard max function (equivalent to infinite inverse temperature). We initialized $\omega_{\text{ij}}^{e/i}$ randomly from a Gumbel distribution with parameters^87,88^ *μ* = 0 and *β* = 0.01.

### Temporal convolutional networks

Following Beniaguev et al.,^13^ we also trained our own version of the temporal convolutional network (TCN) (Figure S5, yellow). As in their main results, we ignore subunits and dendritic morphology and allow all 2,000 excitatory and 200 inhibitory input synapses to engage with each other through four layers of nonlinearities (for reference, Beniaguev et al.^13^ uses seven layers, but their performance reaches near-saturating performance already with four layers, see their Figures S2 and S5). The first layer of our TCN implementation uses 40 temporally convolving filters with a time window of 350 ms. The following layers are feedforward layers with 40 hidden units each. Leaky rectified linear nonlinearities are used between each layer. In total, this results in a DNN with 31 million trainable parameters. For reference, the 7 layer TCN from Beniaguev et al.^13^ has 9.2 million trainable parameters (value obtained from their openly available source code, note that the higher number of parameters in our implementation was primarily due to our usage of a higher temporal resolution of 0.2 ms compared to the 1 ms of Beniaguev et al.^13^).

## Quantification And Statistical Analysis

### Analysis of the biophysical model’s response

The expected response (Figures S1C-S1E) was calculated as the linear sum of the somatic response to the individual synaptic stimulations. In Figures S1F and S1G the NMDA current was measured in 4 randomly selected synapses in each of the 16 dendritic branches and the measured current was averaged across the 4 synapses targeting the same branch and across 16 repetitions.

Dendritic spikes in Figure S7 were detected by the downward crossing of the threshold *θ* = -8 V/s in the temporal derivative of the local dendritic membrane potential. This way we could avoid false positives due to strong local excitatory inputs in high input resistance branches. Events within 5 ms of a somatic action potential were considered coincident, all other events were isolated. Dendrite-soma coupling was defined as the fraction of somatic events during which the branch also fired a spike as in Rolotti et al.^49^ To study the effect of back-propagating action potentials (BAP) on input integration in the biophysical model we first calculated the average local (B)AP waveform (Figures S9A and S9B, top), and then added it to the local (somatic, Figure S9A; or dendritic, Figure S9B) membrane potential response.

### Spike analysis metrics

For the precision-recall analysis (Figures 2F and S11D), we trained a decoder (single GRU subunit, see below, using squared error loss) to predict v_AP_ (see above) from the subthreshold somatic membrane potential of the biophysical model (v_soma_ or v_noNa_; Figure 2F, black and purple, respectively) or the predicted responses of the cascade models (trained to match v_soma_ or v_noNa_ and v_Na_; Figure S11D, blue and red respectively). For calibration, as an upper bound on realizable performance, we also trained a decoder to predict v_AP_ using v_full_ (Figure 2F, orange). In all cases, spikes were detected by finding the threshold crossings of the true or the predicted v_AP_ with positive derivative, with the threshold at 16 mV, and binary spike trains (no spike vs. one or more spikes) were generated by using a moving time window of 1 ms. Table S3 shows how the precision and recall values were computed based on these binarized spike trains.

For performing ROC analyses (Figures S2, S11A, and S11B), we trained a decoder (again a single GRU subunit) to predict the binary spike train of the biophysical model (again obtained by threshold crossings of v_AP_ with positive derivative) from either the subthreshold response of the biophysical model (v_soma_ or v_noNa_; Figure S2, black and purple, respectively) or the predicted responses of the cascade models (trained to match v_soma_ or v_noNa_ and v_Na_; Figures S11A and S11B, blue and red respectively). We also included binary spike train predictions derived from the predictions of a DNN (temporal convolutional networks, TCN, Figures S11A and S11B yellow; following Beniaguev et al.,^13^ see below). As above, for calibration, we also trained a decoder using v_full_ (Figure S2, orange). Importantly, as we trained these decoders with a cross-entropy loss, their output was a predicted instantaneous spiking probability. We then thresholded these predicted spiking probability traces (also only considering threshold crossings with positive derivative) to obtain a predicted spike train, and the corresponding true and false positive rates (Table S3), for a given threshold. We obtained ROC curves by systematically varying this threshold.

The coincidence factor^46^ (Figure 2G) was defined as:

(Equation 21) $$\text{Γ}=\frac{1}{N}\frac{N_{\text{coinc}\;}-\langle N_{\text{coinc}\;}\rangle }{1/2\left(N_{\text{ref}}+N_{\text{pred}\;}\right)}$$

with

(Equation 22) $$N=1-2f_{\text{pred}\;}\text{Δ}$$

where *N*_ref_ is the number of spikes in the reference spike train, *N*_pred_ is the number of spikes in the predicted spike train, and *N*_coiric_ is the number of coincident spikes between the two spike trains using a Δ = 4 ms window around the reference spikes. For these purposes, spike trains were defined based on true or predicted v_AP_, as for the precision-recall analysis (see above). 〈*N*_coinc_〉 = 2 *f*_pred_ Δ *N*_ref_ is the expected number of coincidences generated by a homogeneous Poisson process with same spike rate as the predicted spike train, *f*_pred_ = *N*_pred_/*T* (where *T* is the duration over which the spike trains are recorded).

Spike rate cross correlation analysis^13^ (Figure S11C) was performed between the reference and predicted spike trains (defined as for the ROC analyses, with a detection threshold for the predicted spike probability at 0.04) by calculating the spike rate of the predicted spike train conditioned upon the spikes of the reference spike train within a ± 20 ms time window.

Dendritic spike rates (Figure 2C) were calculated by thresholding the dendritic voltages recorded at the synaptic clusters of the biophysical CA1 model neuron with dendritic Na^+^ channels (Table S2). Specifically, dendritic spikes were detected at each positive crossing of a threshold at – 10 mV.

### Dissimilarity analysis

Representational similarity analysis^52^ allowed us to directly compare the synaptic organization of different model architectures without requiring any structural similarity between them (biophysical or cascade, described by entirely different variables and parameters, or cascades of different structure or model class), as long as there was a correspondence between their synapses. We performed the analysis in two steps. First, we calculated a synaptic dissimilarity matrix for each architecture separately. This matrix was defined as the dissimilarity between any pairs of inputs according to some measure, as specified below. Second, we compared a pair of synaptic dissimilarity matrices using the Pearson correlation coefficient between their corresponding upper triangular entries.

We constructed synaptic dissimilarity matrices using the architecture either defined by the biophysical model or by a cascade model (separately for models fitted to v_Na_ and v_noNa_). For each cascade model, we defined two synaptic dissimilarity matrices, one based on the optimized subunit assignments (Figure 7B, middle and bottom row, right), and another using the optimized synaptic weights (Figure 6C, middle and bottom row, left). The subunit-based dissimilarity matrix was defined by the optimized subunit assignments (Equation 19):

(Equation 23) $$D_{ll^{\prime }}^{s}=1-\delta \left(\underset{n}{argmax}C_{nl}^{\text{syn,e/i}\;},\underset{n}{argmax}C_{nl^{\prime }}^{\text{syn,e/i}}\right)$$

where *δ*(·, ·) is the Kronecker delta.

Elements of the weight-based dissimilarity matrix were calculated for all pairs of excitatory synapses independent of whether they targeted the same or different subunits, as

(Equation 24) $$D_{ll^{\prime }}^{w}=\left|w_{l}^{\text{syn},e}-w_{l^{\prime }}^{\text{syn,e}}\right|$$

where $w_{l}^{\text{syn,e}}$ is the scalar weight associated with input / (Equation 7).

In the biophysical model, we also constructed two dissimilarity matrices: one based on the somatic distances of the synapses (Figure 7B, top left) and one based on the functional clustering of the synapses on the dendritic tree (Figure 7B, top right). We calculated the somatic distance-based dissimilarity matrix as:

(Equation 25) $$D_{ll^{\prime }}^{d}=\left|d_{l}-d_{l^{\prime }}\right|$$

where *d_l_* is the path distance of synapse *l* from the soma along the dendritic tree. For the clustering-based dissimilarity matrix, we used the following distance measure:

(Equation 26) $$D_{ll^{\prime }}^{c}=1-\delta \left(c_{l},c_{\ell^{\prime }}\right)$$

where *c_l_* is the identifier of the input cluster to which (clustered) synapse *l* is assigned.

To compare the dissimilarity matrices obtained from the cascade model to the dissimilarity matrices of the biophysical model, we orthogonalized the clustering-based dissimilarity matrix, ⅅ^*c*^, relative to the somatic distance-based dissimilarity matrix, ⅅ^*d*^. This way, we could remove the influence of somatic distance on the synaptic clustering in the biophysical model. Specifically, we collected the elements of the clustering-based dissimilarity matrix into vector **M**^clust^. Similarly, we formed another vector, **M**^dist^, from the corresponding elements of the distance-based dissimilarity matrix. Then the orthogonalized values were defined as

(Equation 27) $$M^{\text{clust}⊥}=M^{\text{clust}}-M^{\text{dist}}\frac{\langle M^{\text{clust}},M^{\text{dist}}\rangle }{\langle M^{\text{dist}},M^{\text{dist}}\rangle }$$

We used the orthogonalized **M**^clust⊥^ instead of the original clustering-based dissimilarity matrix for all subsequent calculations.

Note that while the elements of the distance- and the weight-based dissimilaritiy matrices are positive real numbers, the subunitbased synaptic dissimilarity matrix is binary and the elements of the clustering-based dissimilaritiy matrix became non-binary only due to the orthogonalization.

In Figure 7D we compared the synaptic dissimilarity matrices of the same cascade model (v_noNa_ or v_Na_) obtained after independently optimizing the synaptic organization of the model on different sets of training data (red and blue), or compared the synaptic dissimilarity matrices of the two different models optimized on the same training data (purple). In Figure 7E we compared the synaptic dissimilarity matrices of the biophysical model to that of the cascade models. When the comparison of two dissimilarity matrices included a clustering- or a subunit-based dissimilarity matrix, we calculated the Pearson correlation between the elements of the matrices corresponding to the 240 clustered synapses. Otherwise, all excitatory synapses were used for the comparison.

### Statistical tests

The details of all statistical tests are included in the captions of the corresponding figures.

## Supplementary Material

Supplementary Information

## Highlights

- The input-output transformation of CA1 neurons is fundamentally dichotomous
- NMDA signals/dendritic Na^+^ spikes control slow V_m_fluctuations/AP timing
- NMDA signals are explained by a single static nonlinearity
- Dendritic Na^+^ spikes are explained by a cascade of dynamic nonlinearities
