# Supplementary material for: Parallel functional architectures within a single dendritic tree
Source: Cell Rep. Author manuscript; Available in PMC 2023 May 13. (PMC7614531; doi:10.1016/j.celrep.2023.112386)
Supplement: Supplementary Information [file EMS175407-supplement-Supplementary_Information.pdf]

Cell Reports, Volume *42*

Supplemental information

Parallel functional architectures within a single dendritic tree

Young Joon Kim, Balázs B. Ujfalussy, and Máté Lengyel

Supplemental Figures

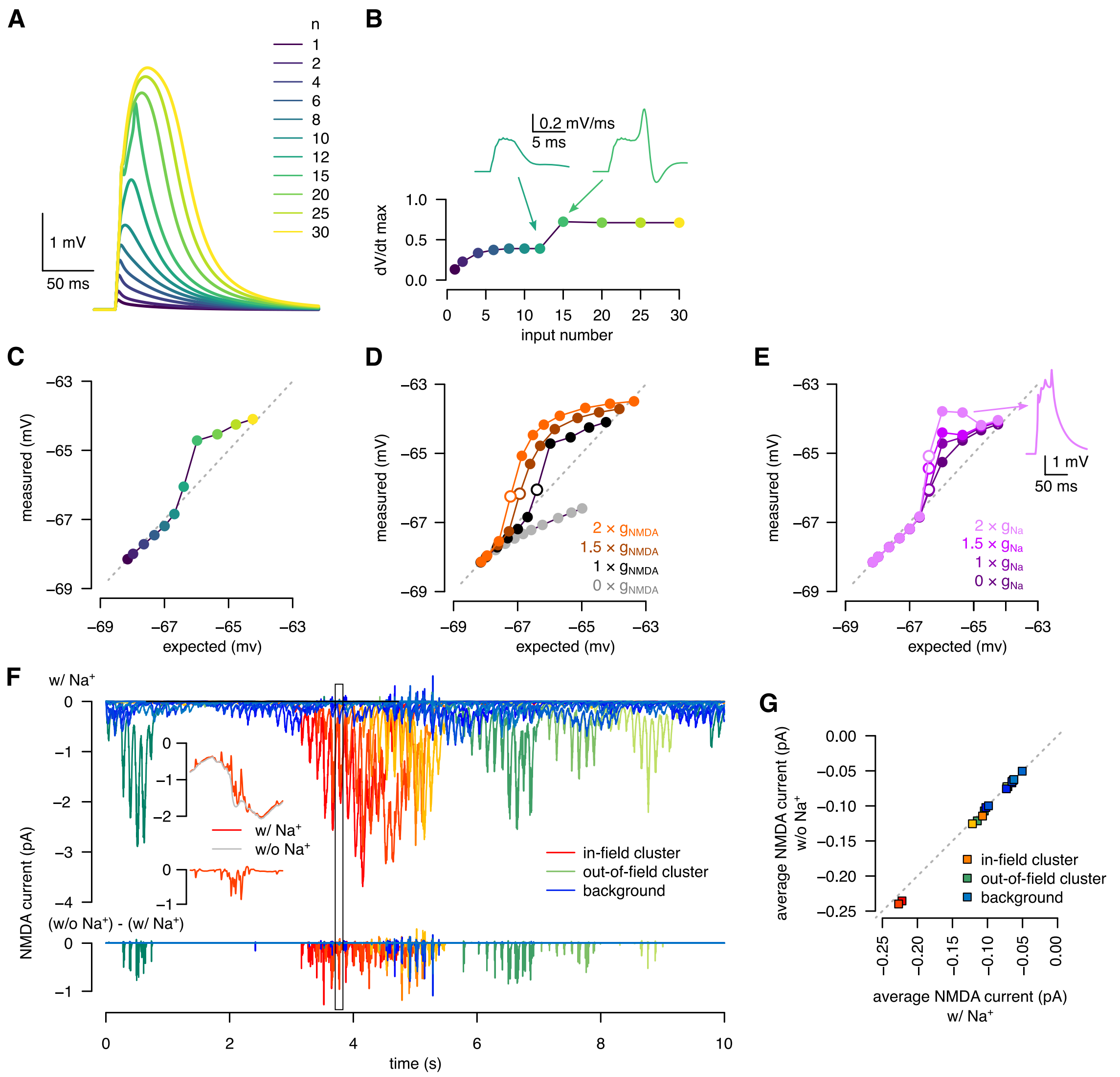

Figure S1. Analysis of Na$^+$- and NMDA-mediated nonlinearities in the biophysical model. Related to Fig. 2.

(**A**) Somatic membrane potential response to the near instantaneous (0.3 ms inster-stimulus interval, ISI) stimulation of an increasing number of synapses (colors) targeting a typical basal dendritic branch with 1 μm inter-synapse distance.

(**B**) Peak of the somatic dV/dt responses as a function of stimulus number (stimulus and colors are the same as in A). Sharp quasi-discontinuity shows threshold for Na$^+$ spike initiation, insets show example dV/dt traces immediately below and above threshold.

(**C**) Measured versus expected somatic response amplitude (stimuli and colors are the same as in A). Expected amplitude is computed using linear summation of voltage responses to individual stimuli.

(**D**) Increasing the maximal NMDA conductance of the synapses from the default $g_{\mathrm{NMDA}} = 0.8\,\mathrm{nS}$ to $1.6\,\mathrm{nS}$ (shades of orange) decreases the *threshold* (empty circle) of the nonlinearity from 12 to 6 inputs, which is substantially lower than the threshold measured experimentally [S1]. Removing the NMDA component entirely renders integration sublinear (grey).

(**E**) Increasing the density of the dendritic voltage-dependent Na$^+$ channels from the default $g_{\mathrm{Na}} = 0.03\,\mathrm{S/cm}^2$ to 0.045 increases the *amplitude* of the supralinear response without changing its threshold (empty circles). Further increasing the dendritic Na$^+$ conductance introduces complex, multi-phase responses (inset, light purple) not observed experimentally [S1, S2]. See also Fig. S6 for an analyses of cascade models fitted to the biophysical model with changed dendritic NMDA and Na$^+$ conductances.

(**F**) Top: Average (across 16 repetitions and 4 randomly selected synapses targeting each branch) NMDA receptor mediated synaptic current in 16 different dendritic branches as a function of simulation time during *in vivo*-like input conditions (presynaptic inputs are shown in Fig. S7A). Branches shown either receive clustered inputs (orange-red: in-field clusters, i.e. maximal input activation overlaps with somatic firing; shades of green: out-of-field clusters, i.e. maximal cluster activity is outside of the location of somatic place field) or only non-clustered, background excitation (blue). Bottom: Change in the NMDA current after blocking all voltage dependent Na$^+$ conductances in dendrites. Inset shows close-up of the period indicated by the rectangle in the main plot. For clarity, the trace for only one of the branches is shown (red), and we included the corresponding trace with dendritic Na$^+$ conductances blocked (top, gray).

(**G**) Temporal average of NMDA currents in the 16 branches shown in panel F with (x-axis) and without (y-axis) dendritic voltage dependent Na$^+$ conductances. Data in **F**-**G** are from the 4-cluster arrangement.

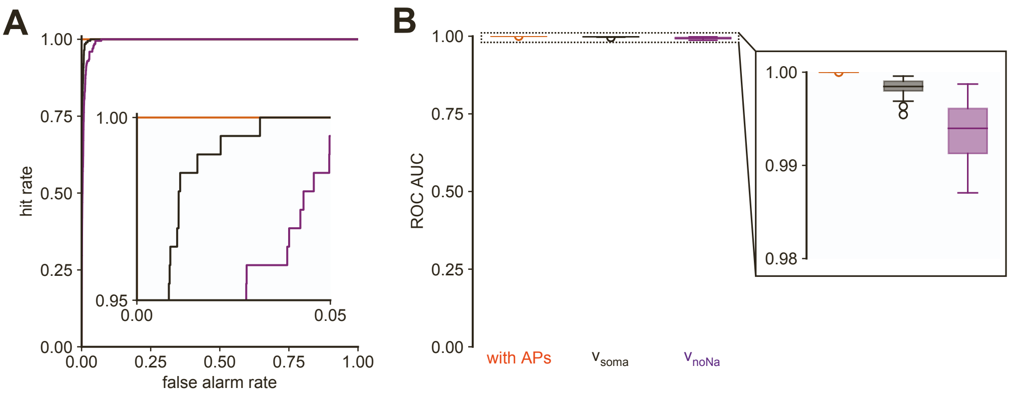

Figure S2. Analysis based on receiver operating characteristic curves overestimates somatic action potential prediction accuracy. Related to Fig. 2.

(**A**) Receiver operating characteristic (ROC) curves obtained for predicting somatic action potentials as in Fig. 2F-G, using a decoder on either the full (orange; v_{full}) or the subthreshold somatic membrane potential with (black; v_{soma}) or without (purple; v_{noNa}) dendritic Na^+ spikes.

(**B**) Areas under curves (AUCs) for the ROCs shown in **A**.

Box plots in **B** show median (horizontal line), 25th & 75th percentiles (box), $\pm$1.5 inter-quartile ranges (whiskers), and outliers (circles) across 20 test trials. Data are from the 4-cluster arrangement.

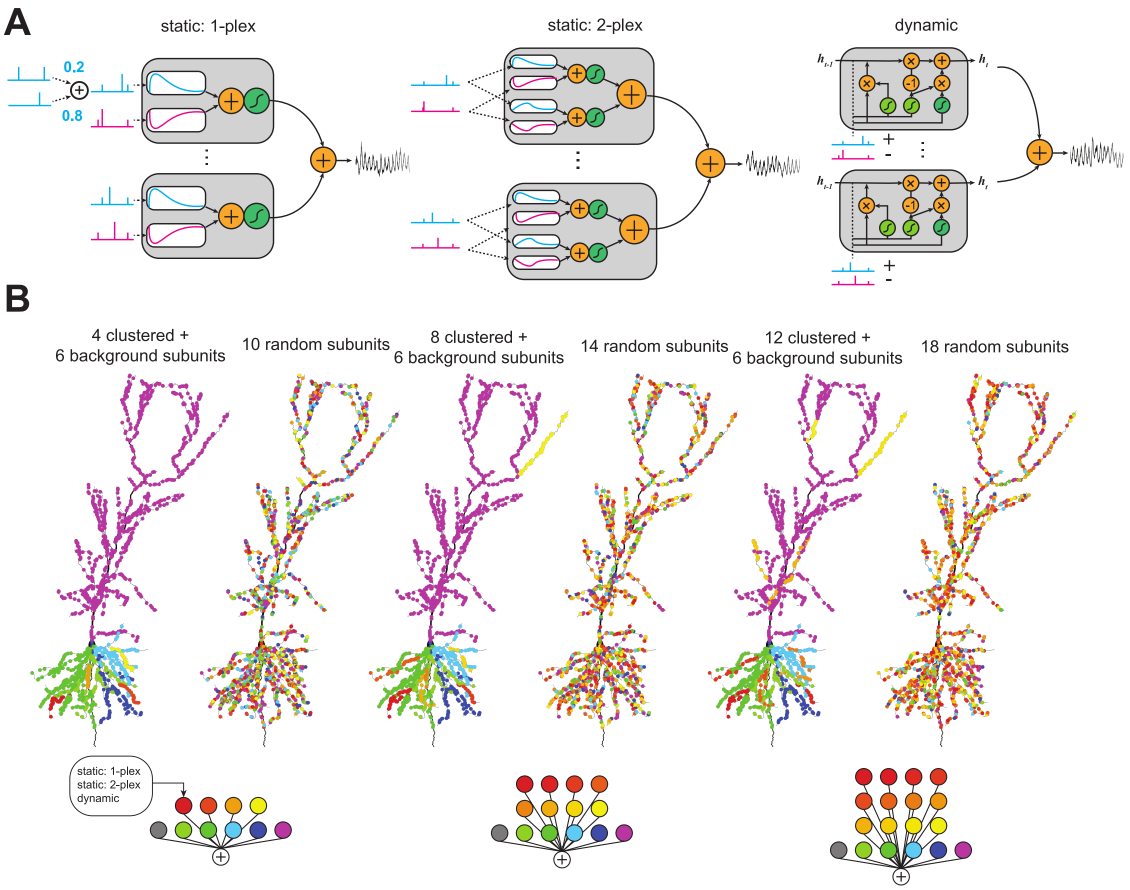

Figure S3. Schematic of models, connectivities, and functional architectures. Related to Fig. 3.
(**A**) Schematic of a pair of 1-plex static (left), 2-plex static (middle), and dynamic subunits (right).
(**B**) Schematics of the different functional architectures used in the paper. Synapses on the biophysical model (top) are colored by the identity of the subunit processing their inputs in the cascade models (bottom). For the 'correct' (first, third, fifth) connectivities (used in Fig. 3 to Fig. 6), hot colors represent the subunits receiving the clustered excitatory inputs (and inhibitory inputs arriving on the same dendritic branches) while cold colors represent subunits receiving the background excitatory inputs (and inhibitory inputs arriving on the same dendritic branches). Inhibitory inputs targeting the soma of the biophysical model were placed on a separate leaf subunit (grey). Finally, the outputs of all leaf subunits were integrated by a single, linear subunit (cross) serving as the output of the cascade model. For the random connectivity (Fig. 6B) synapses are assigned randomly to different subunits.

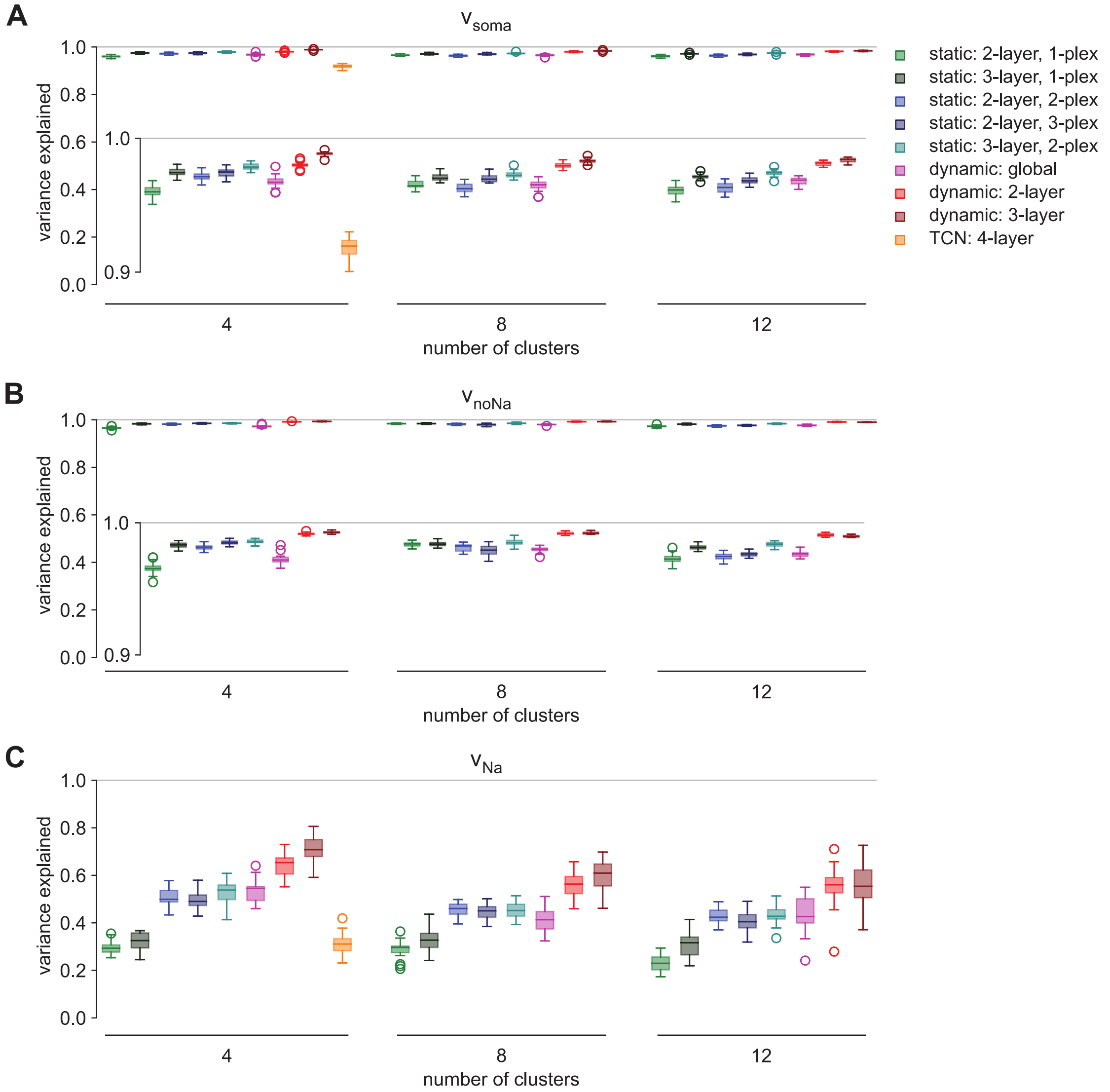

Figure S4. Performance of alternative architectures. Related to Fig. 3.
Cross-validated performance (variance explained) of cascades with a variety of different architectures (including those shown in the main text, light green, blue, and red) predicting v_{soma} (**A**; cf. Fig. 3B), v_{noNa} (**B**; cf. Fig. 4B, left) and v_{Na} (**C**; cf. Fig. 4B, right) for the three cluster arrangements (x-axis, see also Fig. 2B). Note that increasing complexity (more layers, or higher degree of multiplexing) within each model class (static, static with multiplexing, dynamic) only leads to marginal improvements in performance. A densely connected unitary (and static) 'temporal convolutional network'-based architecture [S3] (TCN, orange, see also Fig. S5) shows inferior test error for predicting both v_{soma} (A) or v_{Na} (C).
Box plots show median (horizontal line), 25th & 75th percentiles (box), $\pm$1.5 inter-quartile ranges (whiskers), and outliers (circles) across 20 test trials.

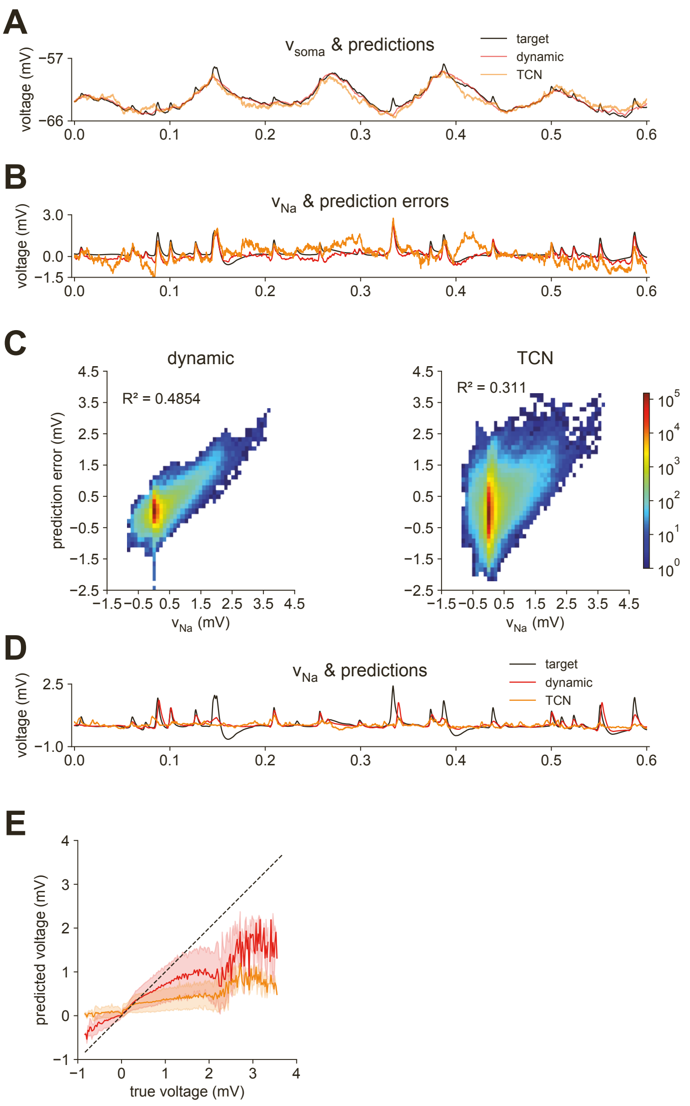

Figure S5. Predictions of a densely connected unitary temporal convolutional network-based architecture [S3] (TCN). Related to Fig. 3.

(**A**) Sample somatic membrane potential trace of the biophysical model neuron (black, replotted from Fig. 3A, black) with predicted v_{soma} traces from the dynamic cascade model (red, replotted from Fig. 3A, red) and a 4-layer TCN (orange).

(**B**) Sample traces of the Na^+ differential voltage (v_{Na}, black) and prediction error residuals of the dynamic cascade model and TCN (colors as in **A**) during the same trial as shown in **A** (black and red replotted from Fig. 3D).

(**C**) Pearson correlation (R^2) and joint histogram of the dynamic cascade model's (left, replotted from Fig. 3E, left) and the TCN's v_{soma} prediction errors (right) and the Na^+ differential voltage, v_{Na}.

Data are from the 4-cluster arrangement.

(**D**) Sample traces of the Na^+ differential voltage (v_{Na}, black) and v_{Na} predictions from the dynamic cascade model and TCN (colors as in **A**) during the same trial as shown in **A** (cf. Fig. 4A, bottom, black and red).

(**E**) Predicted v_{Na} voltages as a function of the measured v_{Na} for the dynamic cascade model (replotted from Fig. 4C, right) and TCN (colors as in **A**). Lines and shaded areas show mean$\pm$1 s.d. across 20 test trials.

Data are from the 4-cluster arrangement. For quantifying TCN prediction accuracy, see also Fig. S4A and C.

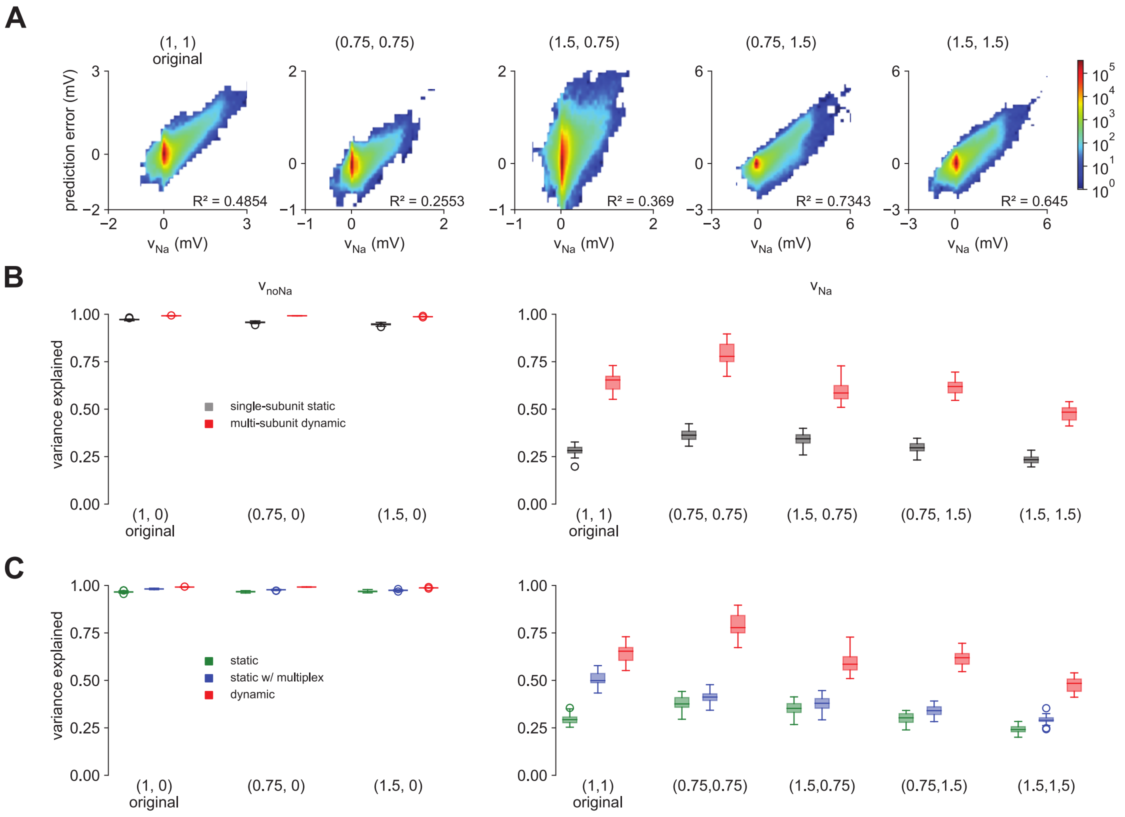

Figure S6. Robustness of main results to variations in Na^+ and NMDA conductances. Related to Fig. 4.
(**A**) Pearson correlation (R^2) and joint histogram of the dynamic cascade model's v_{soma} prediction errors and the Na^+ differential voltage, v_{Na}, for the original channel conductances (left, replotted from Fig. 3E) and for four different combinations of dendritic Na^+ and NMDA maximal conductances (see also Fig. S1). Numbers in parentheses on top of each sub-panel here and on the x-axes of all other panels show multiplicative factors used to change the dendritic NMDA and Na^+ maximal conductances, respectively, relative to the original parameters.
(**B**) Cross-validated performance (variance explained) of the single-subunit static (gray) and multi-subunit dynamic cascades (red) for v_{noNa} (left) and v_{Na} (right) using different dendritic NMDA and Na^+ maximal conductances (numbers in parentheses as in **A**). Note that the Na^+ conductance multipliers are all 0 for v_{noNa} as it is obtained by removing the effects of all dendritic Na^+ conductances.
(**C**) Cross-validated performance (variance explained) of multi-subunit static (green), static with multiplexing (blue), and dynamic (red) cascade models for v_{noNa} (left) and v_{Na} (right) using the same alternative biophysical model parameterizations as in **B**. Data labelled as "original" is replotted from Fig. 4B.
Box plots in **B**-**C** show median (horizontal line), 25th & 75th percentiles (box), ± 1.5 inter-quartile ranges (whiskers), and outliers (circles) across 20 test trials. Data are from the 4-cluster arrangement.

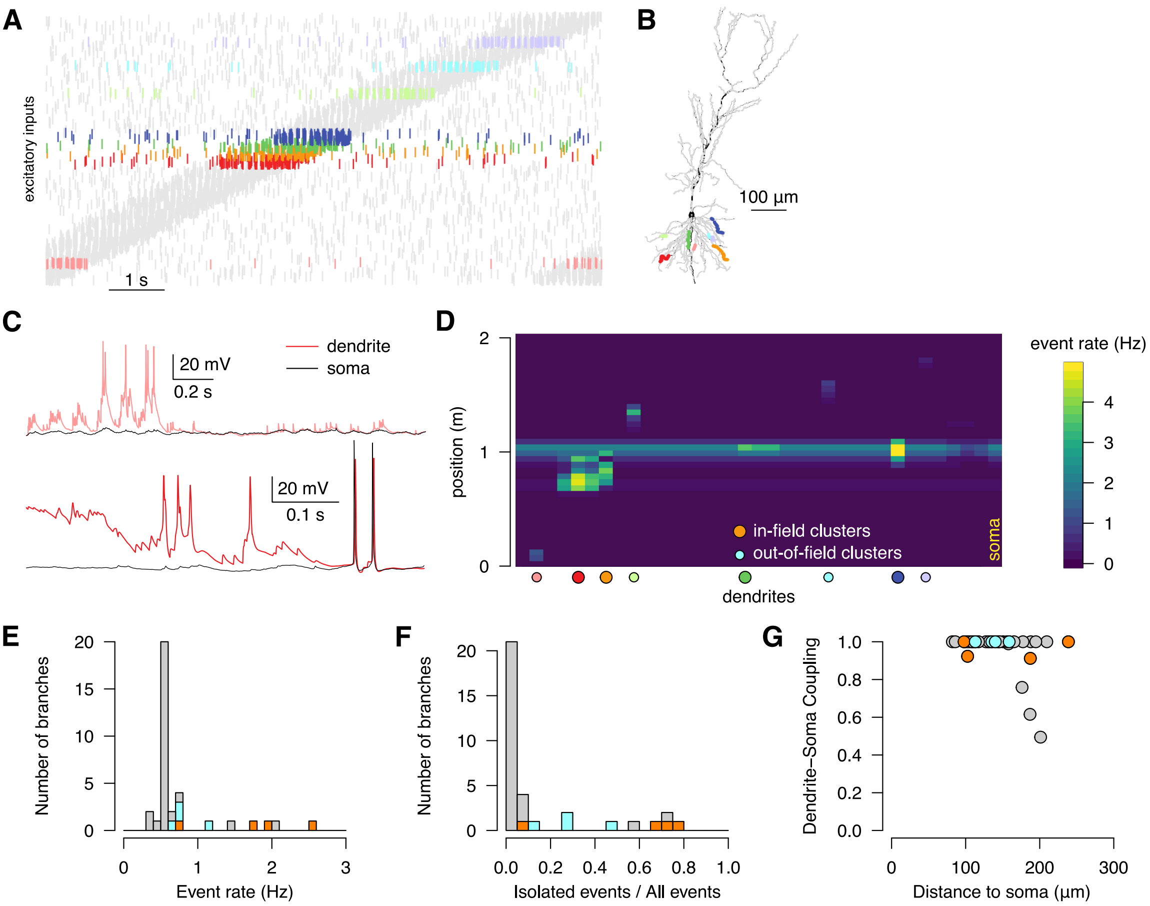

Figure S7. Local dendritic spikes inside and outside the somatic place field in the biophysical model. Related to Fig. 4.
(**A**) Spike raster plots for one 10-second trial showing spikes at 2000 excitatory input synapses as in Fig. 2A. Note the 4 smaller input clusters (pale colors) in addition to the larger clusters used for the simulation shown in the main text (bright colors). These smaller clusters receive inputs whose activity peaks outside of the somatic place field, which is around the middle of the track (as determined by the activity of inputs impinging the larger clusters).
(**B**) Schematic showing the locations of input synapses along the dendritic tree as in Fig. 2B. Again, note the 4 smaller input clusters (pale colors) in addition to those used for the main simulations (bright colors).
(**C**) Examples of simultaneous dendritic (pink and red, colors as in **A**-**B**) and somatic (black) membrane potential traces show dendritic Na^+ spikes and NMDA plateaus in the absence of somatic spiking.
(**D**) Rate of spiking events (STAR Methods) in 34 basal dendritic branches (columns) and in the soma (rightmost column) at different spatial locations along the simulated maze (rows). Small and large colored circles along x-axis indicate cluster identity of corresponding branches receiving in- or out-of-field inputs, respectively (colors as in **A**-**C**).
(**E**) Histogram of the average event rate across dendritic branches. Gray, orange and cyan colors indicate branches receiving background, in- and out-of-field clustered inputs, respectively (see also legend in **D** for colors).
(**F**) Histogram of the proportion of isolated dendritic events (i.e. without a simultaneous somatic action potential) across dendritic branches. Colors as in **E**.
(**G**) Dendrite-soma coupling (STAR Methods; see Rolotti et al. (2022) [S4]) as a function of the distance of the branch from the soma. Colors as in **E**.

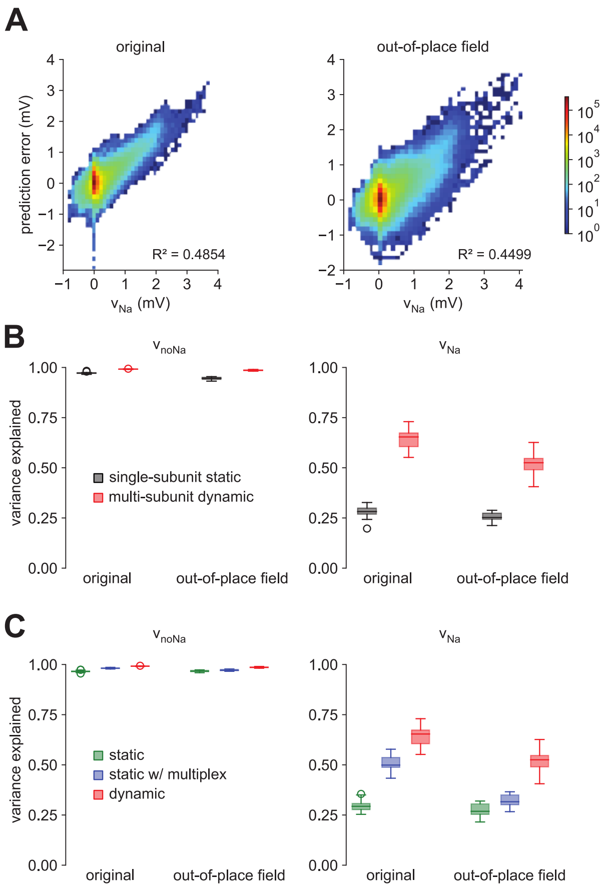

Figure S8. **Robustness of main results with out-of-place field inputs.** Related to Fig. 4.
(**A**) Pearson correlation (R^2) and joint histograms of the the dynamic cascade model's v_{soma} prediction errors and the Na^+ differential voltage, v_{Na} using a biophysical model with original inputs (left, replotted from Fig. 3) and out-of-place field clustered inputs (right; see also Fig. S7).
(**B**) Cross-validated performance (variance explained) of the single-subunit static (gray) and multi-subunit dynamic cascade models (red) for v_{noNa} (left) and v_{Na} (right) with original inputs (replotted from Fig. S6B) and with out-of-place field clustered inputs (see also Fig. S7) .
(**C**) Cross-validated performance (variance explained) of multi-subunit static (green), static with multiplexing (blue), and dynamic (red) cascade models for v_{noNa} (left) and v_{Na} (right) with original (replotted from Fig. 4B) and out-of-place field clustered inputs (see also Fig. S7).
Box plots in **B**-**C** show median (horizontal line), 25th & 75th percentiles (box), $\pm$1.5 inter-quartile ranges (whiskers), and outliers (circles) across 20 test trials. Data are from the 4-cluster arrangement (original) with an additional 4 out-of-place field clusters for the new simulations.

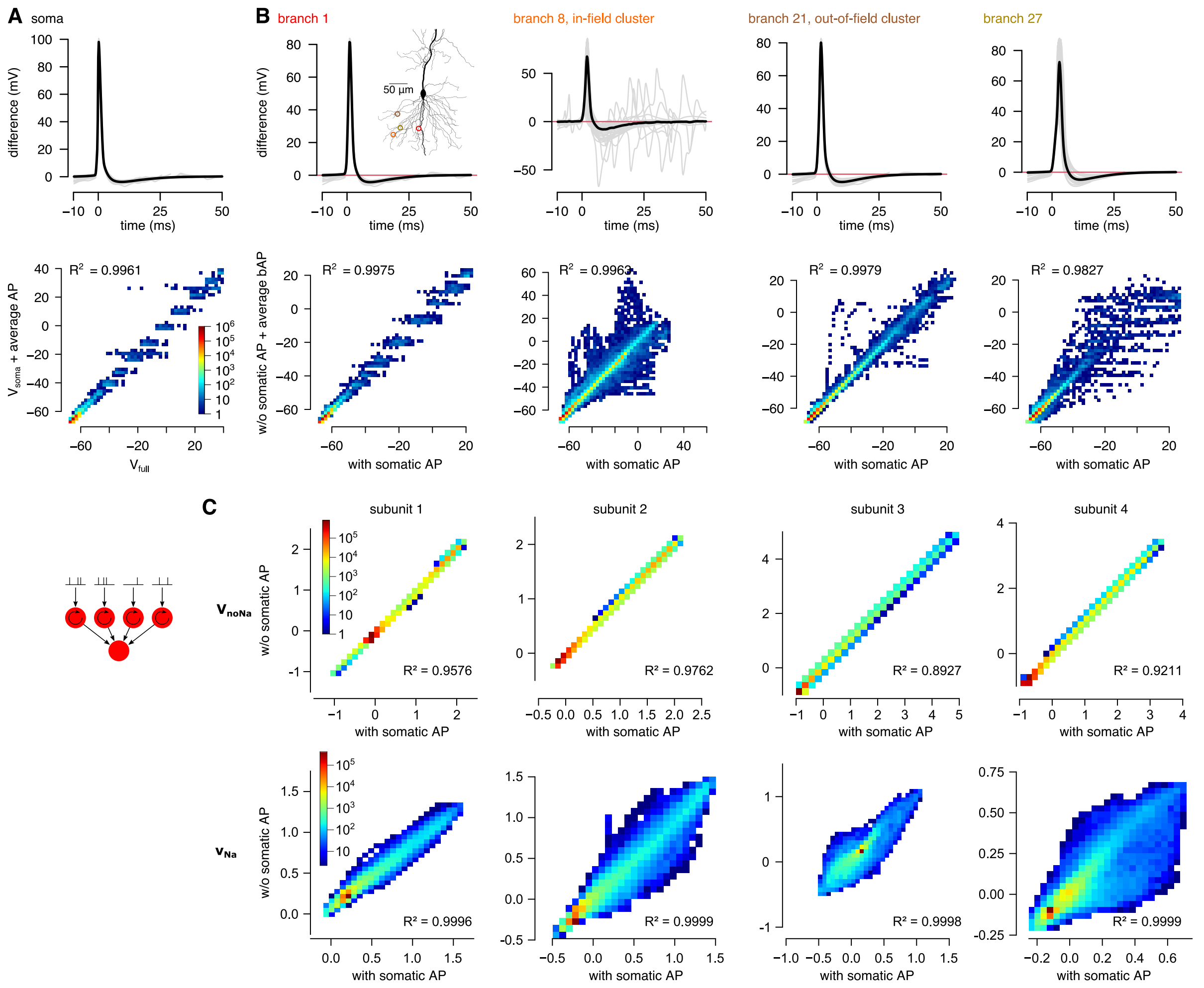

Figure S9. Effects of backpropagating action potentials (bAPSs) on dendritic integration. Related to Fig. 4.
(**A**) Top: Spike triggered average difference (thick black curve) between the biophysical model's somatic membrane potential with action potentials (v_{full}) and without somatic Na^{+} channels (v_{soma}). Grey lines show individual traces. Bottom: Pearson correlation (R^2) and joint histograms of v_{full} (x-axis) and v_{soma} + average action potential from top panel (y-axis). A high correlation indicates a simple, stereotypical additive contribution of somatic action potential generation to the somatic membrane potential.
(**B**) Similar to panel A for 4 different dendritic branches (inset on left), showing somatic spike-triggered bAPs (top) and their contributions to the local dendritic membrane potential. A high correlation in bottom panels indicates a simple, stereotypical additive contribution of bAPs to the local dendritic membrane potential, and thus no discernible effect on dendritic integration.
(**C**) Pearson correlation (R^2) and joint histograms of the the dynamic cascade model's (inset on left) subunit outputs for predicting v_{noNa} (top) and v_{Na} (bottom) with (x-axis, i.e. training on v_{full}) and without incorporating bAPs (y-axis, i.e. training on v_{soma}). Data are from the 4-cluster arrangement with out-of-place field clustered inputs (Fig. S7).

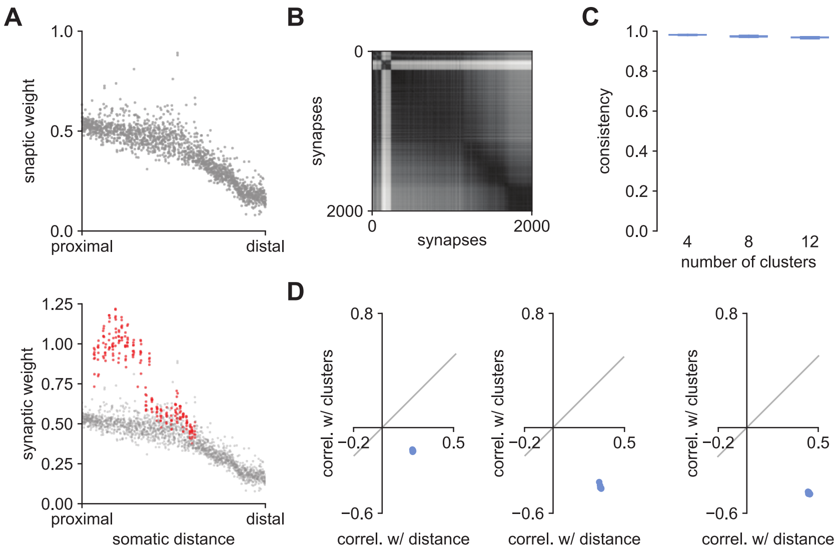

Figure S10. Optimized synaptic weights for single-subunit static model fitted to v_{noNa}. Related to Fig. 6.

(**A**) Optimized weights of background (top) and clustered synapses (bottom, red) in the single-subunit v_{noNa} architecture against their somatic distances in the biophysical model (cf. Fig. 6D, left). Background synapses are shown in grey in bottom panel for reference.

(**B**) Synaptic weight-based dissimilarity matrix of the single-unit v_{noNa} architecture (averaged across fits to different training data sets, cf. Fig. 7B middle left matrix).

(**C**) Consistency of the synaptic organizations for the single-subunit v_{noNa} architectures (Pearson correlation coefficient between pairs of synaptic weight-based dissimilarity matrices matrices obtained for fits to different training data sets of the v_{noNa} voltage signal) for the three cluster arrangements (cf. Fig. 7C, light blue).

(**D**) Correlation (Pearson correlation coefficient) of the synaptic organizations for the single-subunit v_{noNa} architecture with the somatic distances (x-axis) and dendritic clustering of synapses (y-axis; cf. Fig. 7D, light blue) for the three cluster arrangements. Box plots in **C** show median (horizontal line), 25th & 75th percentiles (box), $\pm$1.5 inter-quartile ranges (whiskers), and outliers (circles) across 5 separate training data sets (not all of these elements are readily visible due to their small sizes, and the lack of outliers). Cross-hairs in **D** show mean$\pm$1 s.d. along each axis across 5 training data sets (not readily visible due to their small sizes). Data in **A**-**B** are from the 4-cluster arrangement.

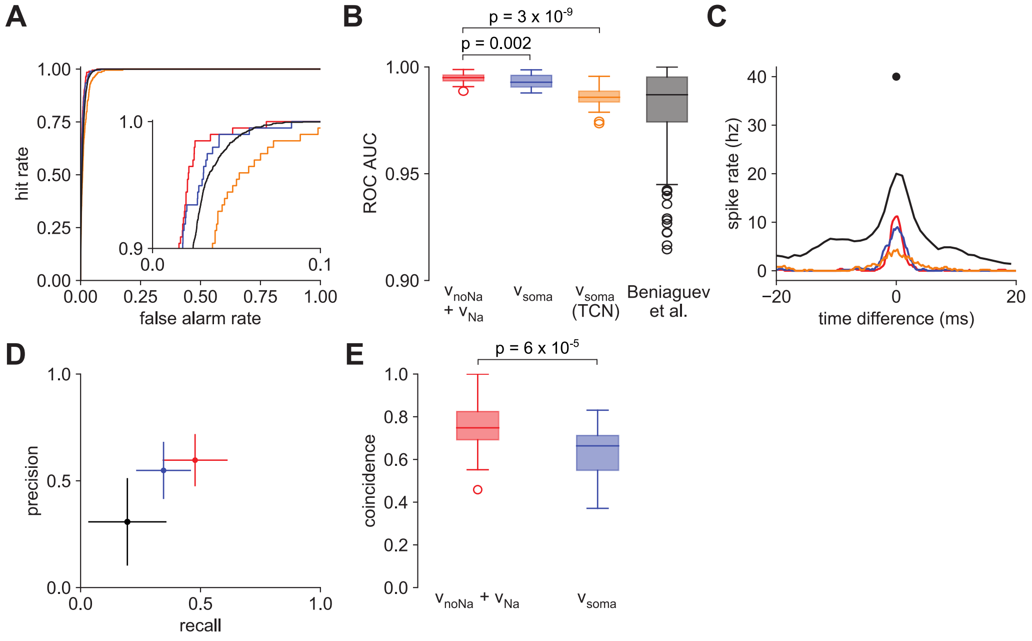

Figure S11. Predicting somatic action potentials with different architectures. Related to STAR Methods.
(**A**) Receiver operating characteristic (ROC) curves obtained for predicting somatic action potentials as in Fig. S2, using a decoder either on the sum of the outputs of the separate cascades fitted to v_{noNa} and v_{Na} (red, weighted sum with optimized coefficients) or on the output of the unitary cascade fitted directly to v_{soma} (blue). For reference, we also show the ROC curve associated with a densely connected unitary architecture (TCN, see also Fig. S5) trained to predict the somatic APs of our CA1 model neuron (orange) and the same TCN model trained to predict spikes of a neocortical L5 model (black, reanalysed from Beniaguev et al. (2021) [S3], cf. their Fig. 2D).
(**B**) Areas under curves (AUCs) for the ROCs shown in **A**.
(**C**) Spike rate cross correlation between the biophysical model and the outputs of the abstract models (colors as in **A**, cf. Fig. 2F of Ref. S3).
(**D**) Precision versus recall of somatic action potential prediction as in Fig. 2F, using a decoder for the cascade models separately fitted to v_{noNa} and v_{Na} (red, cf. red in **A**-**C**) and a unitary cascade model fitted directly to v_{soma} (blue, cf. blue in **A**-**C**). For reference, the performance of the Beniaguev et al, (2021) model [S3] is also shown (black).
(**E**) Same as **D** but quantifying decoding accuracy with the spike coincidence metric of Naud et al. (2014) [S5] as in Fig. 2G.
Box plots in **B** and **E** show median (horizontal line), 25th & 75th percentiles (box), $\pm$1.5 inter-quartile ranges (whiskers), and outliers (circles) across 20 test trials. Statistical comparisons shown in **B** and **E** use two-tailed paired t-tests (for n=20 test trials). Cross-hairs in **D** show mean$\pm$1 s.d. along each axis across 20 test trials. Data are from the 4-cluster arrangement.

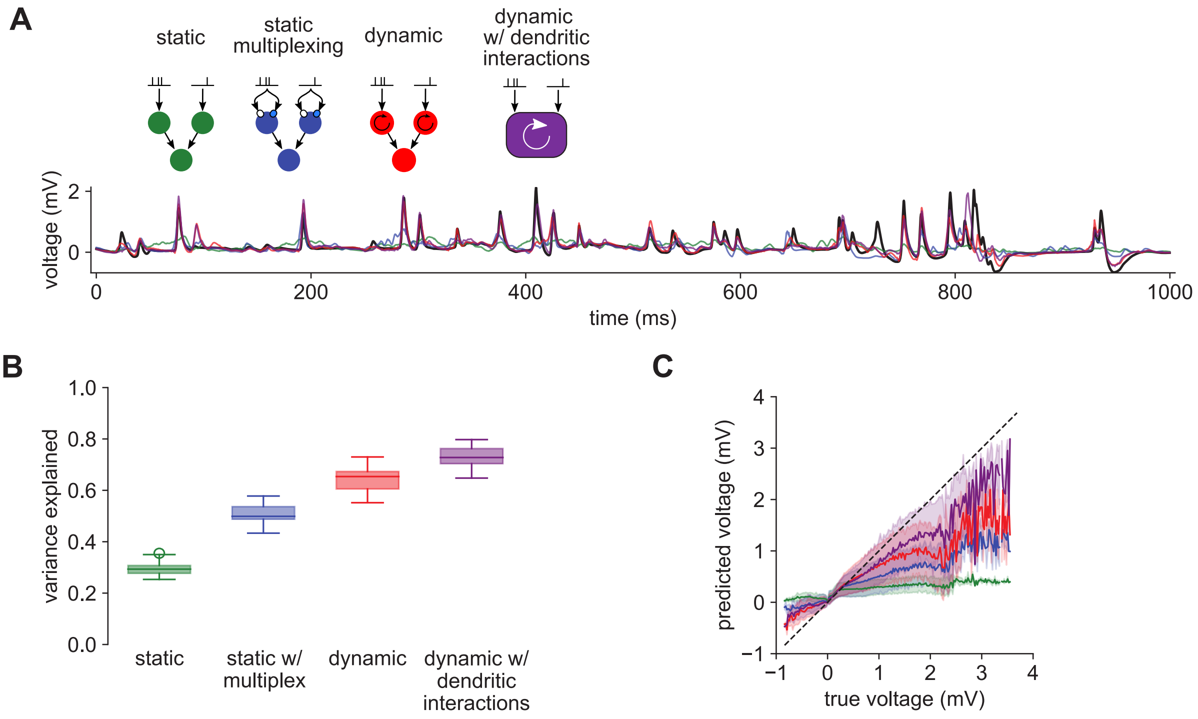

Figure S12. Dendritic interactions contribute to somatic subthreshold voltage. Related to STAR Methods.
(**A**) Sample v_{Na} trace of the biophysical model neuron (black) with the predicted voltages of four different cascade model types. Inset shows schematic architectures of cascade models: static, static with multiplexing, and dynamic cascades used throughout the paper, in which subunits independently feed into the output (respectively green, blue, and red as in the inset of Fig. 4A), and a dynamic model that can incorporate arbitrary interactions between subunits (purple). Although the dynamical model with interactions was implemented technically as a single subunit, it was fundamentally more flexible than the single-unit dynamical architecture shown in Fig. S4 ("dynamic: global", purple): the former received the spike trains of each subunit as a separate input (thus retaining the notion of 'subunits'), while the latter received a single input which combined all input spike trains (see also STAR Methods).
(**B**) Cross-validated performance (variance explained) of the four cascade types (colors as in **A)** for v_{Na}.
(**C**) Predicted voltage as a function of the measured voltage for the four cascade types (colors as in **A**).
Data are from the 4-cluster arrangement. For ease of comparison in **B** and **C**, data for the three cascade types used in the main text (green, blue, and red) are replotted from Fig. 4B and C (right), respectively.

**feature**	$\mathbf{v_{noNa}}$	$\mathbf{v_{Na}}$	figure
controls	subthreshold fluctuations	somatic action potential timing	Fig. 2
type of subunit(s)	static	dynamic	Fig. 4
number of subunits	single	multiple	Fig. 5
defining computational properties	weights of synapses	subunit assignment of synapses	Fig. 6, Fig. 7
defining biophysical properties	somatic distance	dendritic clustering	Fig. 6, Fig. 7

Table S1. Summary of differences between $\mathbf{v_{noNa}}$ and $\mathbf{v_{Na}}$ architectures. Related to Fig. 7.

figures	v_{full}	v_{soma}	v_{noNa}	$v_{AP} = v_{full} - v_{soma}$	$v_{Na} = v_{soma} - v_{noNa}$
2C		✓ (green)			
2D		✓ (black) ✓ (grey)			
3E	✓ (black)	✓ (black) ✓ (grey)	✓ (black)		✓ (black)
3F-G	✓ (teal)	✓ (teal)	✓ (teal)	✓ (orange)	
3A		✓ (black) ✓ (red)			
3B-C		✓ (orange) ✓ (red)			
3D		✓ (red)			✓ (black)
3E		✓ (red)			✓ (orange)
4A		✓ (black)	✓ (black) ✓ (red)		✓ (black) ✓ (red)
4B-C			✓ (orange) ✓ (red)		✓ (orange) ✓ (red)
4D-E			✓ (red)		✓ (red)
5B			✓ (orange) ✓ (red)		✓ (orange) ✓ (red)
6B			✓ (orange) ✓ (red)		✓ (orange) ✓ (red)
6C-D			✓ (red)		✓ (red)
6E		✓ (grey)			✓ (red)
7C-D			✓ (red)		✓ (red)
S2A-B	✓ (teal)	✓ (teal)	✓ (teal)	✓ (orange)	
S4A		✓ (orange) ✓ (red)			
S4B			✓ (orange) ✓ (red)		
S4C					✓ (orange) ✓ (red)
S5A		✓ (black) ✓ (red)			
S5B		✓ (red)			✓ (black)
S5C		✓ (red)			✓ (orange)
S5D					✓ (black) ✓ (red)
S5E					✓ (orange) ✓ (red)
S6A		✓ (red)			✓ (orange)
S6B,D			✓ (orange) ✓ (red)		
S6C,E					✓ (orange) ✓ (red)
S8A-B		✓ (red)			✓ (orange)
S8C,E			✓ (orange) ✓ (red)		
S8D,F					✓ (orange) ✓ (red)
S10A-E			✓ (red)		
S11A-E		✓ (red)	✓ (red)	✓ (orange)	✓ (red)
S12A					✓ (black) ✓ (red)
S12C-D					✓ (orange) ✓ (red)

Table S2. Summary of biophysical and cascade models used in figures. Related to STAR Methods.
✓ (black) indicates figure panels that have the indicated models' *somatic* membrane potential output explicitly plotted. ✓ (grey) indicates panels that have the biophysical models' *dendritic* membrane potential explicitly plotted. ✓ (teal) indicates panels that use the indicated models' somatic outputs for various calculations other than for assessing model fits (e.g. spike prediction analysis). ✓ (green) indicates panels that use the indicated models' subunit outputs for various calculations other than for assessing model fits (e.g. spike prediction analysis). ✓ (orange) indicates panels that use the indicated models' outputs specifically for assessing model fits (e.g. variance explained). ✓ (red) indicates panels that use cascade models that were fitted to the indicated voltage target. Lighter versions of each check represent instances where the models' *dendritic* voltage traces were used rather than their somatic outputs. v_{full} is the somatic membrane potential including action potientials. v_{soma} is the subthreshold somatic membrane potential with somatic and axonal Na^{+} channel conductance set to 0 in the biophysical model. v_{noNa} is the subthreshold somatic membrane potential in the absence of axonal, somatic and dendritic Na^{+} channels. $v_{AP} = v_{full} - v_{soma}$ and $v_{Na} = v_{soma} - v_{noNa}$.

TP (true positive)	P(prediction=1, true=1)
FN (false negative)	P(prediction=0, true=1)
FP (false positive)	P(prediction=1, true=0)
TN (true negative)	P(prediction=0, true=0)
hit rate (recall)	P(prediction=1 | true=1) = TP / (TP + FN)
false alarm rate	P(prediction=1 | true=0) = FP / (TN + FP)
precision	P(true=1 | prediction=1) = TP / (TP + FP)

Table S3. Spike prediction accuracy measures. Related to STAR Methods.

Supplemental References

S1. A. Losonczy, J. C. Magee, Integrative Properties of Radial Oblique Dendrites in Hippocampal CA1 Pyramidal Neurons, Neuron 50 (2) (2006) 291–307. doi:10.1016/j.neuron.2006.03.016.

S2. A. Losonczy, J. K. Makara, J. C. Magee, Compartmentalized dendritic plasticity and input feature storage in neurons, Nature 452 (7186) (2008) 436–441. doi:10.1038/nature06725.

S3. D. Beniaguev, I. Segev, M. London, Single cortical neurons as deep artificial neural networks, Neuron 109 (17) (2021) 2727–2739.e3. doi:10.1016/j.neuron.2021.07.002.

S4. S. V. Rolotti, H. Blockus, F. T. Sparks, J. B. Priestley, A. Losonczy, Reorganization of ca1 dendritic dynamics by hippocampal sharp-wave ripples during learning, Neuron 110 (6) (2022) 977–991.e4. doi:10.1016/j.neuron.2021.12.017.

S5. R. Naud, B. Bathellier, W. Gerstner, Spike-timing prediction in cortical neurons with active dendrites, Frontiers in computational neuroscience 8 (2014) 90.
